# *De novo* damaging variants associated with congenital heart diseases contribute to the connectome

**DOI:** 10.1038/s41598-020-63928-2

**Published:** 2020-04-27

**Authors:** Weizhen Ji, Dina Ferdman, Joshua Copel, Dustin Scheinost, Veronika Shabanova, Martina Brueckner, Mustafa K. Khokha, Laura R. Ment

**Affiliations:** 1 Departments of Pediatrics, New Haven, CT USA; 2 Obstetrics, Gynecology and Reproductive Sciences, New Haven, CT USA; 3 Radiology and Biomedical Imaging, New Haven, CT USA; 4 Genetics, New Haven, CT USA; 5 Yale Combined Program in Biological and Biomedical Sciences, New Haven, CT USA; 6 Neurology, Yale School of Medicine, 333 Cedar Street, New Haven, CT USA

**Keywords:** Genetics, Paediatric research

## Abstract

Congenital heart disease (CHD) survivors are at risk for neurodevelopmental disability (NDD), and recent studies identify genes associated with both disorders, suggesting that NDD in CHD survivors may be of genetic origin. Genes contributing to neurogenesis, dendritic development and synaptogenesis organize neural elements into networks known as the connectome. We hypothesized that NDD in CHD may be attributable to genes altering both neural connectivity and cardiac patterning. To assess the contribution of *de novo* variants (DNVs) in connectome genes, we annotated 229 published NDD genes for connectome status and analyzed data from 3,684 CHD subjects and 1,789 controls for connectome gene mutations. CHD cases had more protein truncating and deleterious missense DNVs among connectome genes compared to controls (OR = 5.08, 95%CI:2.81–9.20, Fisher’s exact test P = 6.30E-11). When removing three known syndromic CHD genes, the findings remained significant (OR = 3.69, 95%CI:2.02–6.73, Fisher’s exact test P = 1.06E-06). In CHD subjects, the top 12 NDD genes with damaging DNVs that met statistical significance after Bonferroni correction (*PTPN11, CHD7, CHD4, KMT2A, NOTCH1, ADNP, SMAD2, KDM5B, NSD2, FOXP1, MED13L, DYRK1A*; one-tailed binomial test P ≤ 4.08E-05) contributed to the connectome. These data suggest that NDD in CHD patients may be attributable to genes that alter both cardiac patterning and the connectome.

## Introduction

Congenital heart disease (CHD) affects nearly 1% of all births in the United States each year^1,2^, and 20–30% of subjects with CHD have extra-cardiac abnormalities or neuro-developmental disability (NDD)^3–6^. Approximately 1/3^rd^ of these infants will be diagnosed with a severe CHD, or those that require intervention within the first postnatal year, and the incidence of NDD increases with the severity of the CHD^4^. Thus, approximately 20% of children with a mild CHD will have a NDD, while more than 50% of children with severe CHD experience NDDs ranging from developmental delay and intellectual disability to autism spectrum disorder and attention-deficit/hyperactivity disorder (ADHD)^7–15^. Further, although neonates with CHD are at risk for hypoxemia in the pre- and postnatal periods^5,16–22^ and as many as 1/3^rd^ of cases have a genetic syndrome associated with their CHD^23–25^, currently identified risk factors explain less than half of neurodevelopmental outcomes in infants with severe CHD^5,26–31^. Therefore, as genomic data for CHD is becoming more and more available, we sought to explore a genetic basis for NDD in patients with CHD.

“Neural connectivity is the intermediate between molecules and behavior”^32^, and NDDs ranging from developmental delay, intellectual disability and autism to ADHD, depression and schizophrenia have been attributed to alterations in the microstructural and functional networks of the developing brain, or the “connectome”^32,33^. When compared to typically developing controls, magnetic resonance imaging (MRI) studies in over 1/3^rd^ of fetuses with isolated CHD show reduced brain volumes and delayed cerebral maturation^34,35^. Similarly, neural connectivity data demonstrate alterations that mediate behavior in children and adolescents with CHD^36–38^, suggesting that disruption of the connectome perturbs neurocognition in this vulnerable population. Since alterations in neural networks are present in neonates with CHD prior to surgery^39^, these data suggest that changes in neural connectivity could be secondary to mutations in the genome rather than solely due to hemodynamic factors, and recent genomic studies have identified candidate genes common to children with CHD and those with NDD, suggesting a genetic basis for CHD-associated NDD^23,24,40,41^. In these subjects, genetic substitutions affect genes involved in chromatin modification, morphogenesis and transcriptional regulation of neuronal tissues as well as in the heart. *De novo* variants (DNVs) in certain chromatin modifiers, important for fetal brain development^42–45^, account for 14/35 (40%) of variants of patients with both CHD and autism spectrum disorder^40^.

**Figure 1:**
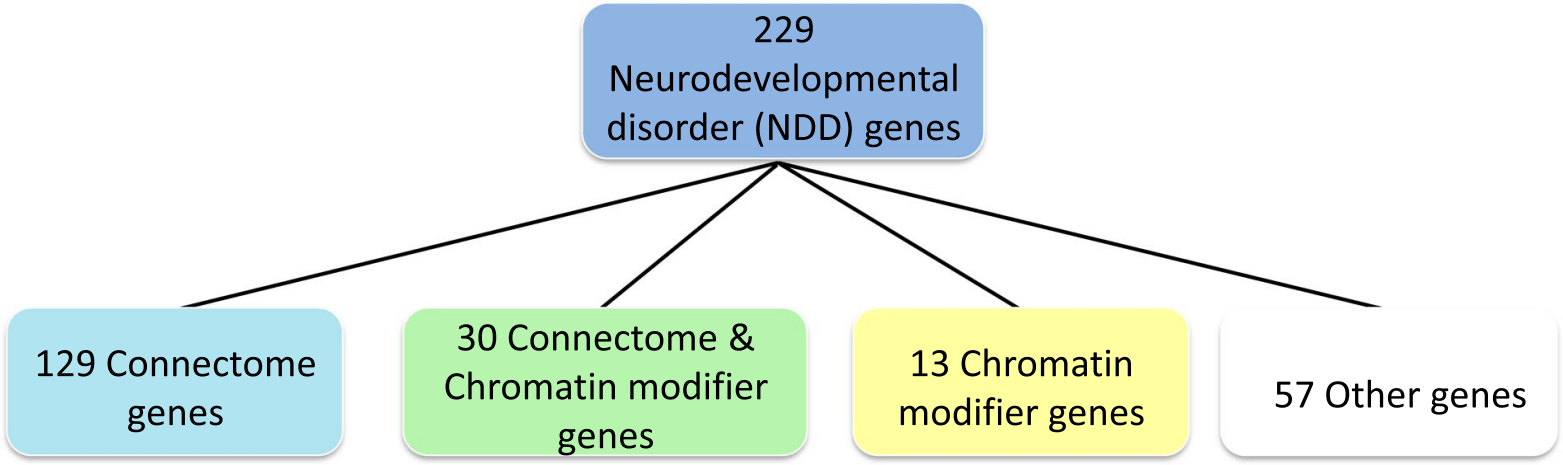
Characterization of the 229 Neurodevelopmental Disorder (NDD) genes. Review of the literature demonstrated that 129 of the published 229 NDD genes contributed to the connectome, 13 were chromatin modifiers, and 30 were chromatin modifiers that contributed to the connectome. Fifty-seven were neither chromatin modifiers nor connectome genes.

As the adult CHD population is significantly expanding and the need to understand the many causes of NDD in these patients is urgently increasing^46^, we tested the hypothesis that NDD in severe CHD patients, or those at highest risk for NDDs, may be attributable to genes that alter both neural connectivity and cardiac patterning. To assess the contribution of DNVs in connectome genes we annotated 229 published NDD genes for connectome status^47^ and analyzed genomic data from 3,684 subjects with CHD and 1,789 controls for connectome gene mutations^40,48^. Genes contributing to the connectome were defined as those necessary for the development, growth and maintenance of neural networks in the developing brain and included those subserving neurogenesis, axonal migration, dendritic development and/or synaptogenesis (Fig. 1)^49–51^. Our second aim was to test the hypothesis that DNVs in chromatin modifiers contributing to the connectome occur more commonly in subjects with CHD than in control subjects. We defined chromatin modifiers as genes that alter DNA or protein in chromatin by the covalent addition or removal of chemical groups^52^.

## Results

To assess the contribution of DNVs within CHD to the developing connectome, we studied 3,684 unique, previously published, proband/parent trios with CHD and exome sequencing data. The CHD trios consist of affected probands and their two unaffected parents. These included 1039 probands as part of the multi-center/multi-study cohort from the Deciphering Developmental Disorders Project (“DDD Plus Study”)^48^ and 2,645 probands from the Pediatric Cardiac Genomics Consortium (PCGC) study (see Supplementary Table S1 for comparison of the study cohorts)^40^. Three hundred twenty six probands in the DDD Plus Study were PCGC cases, and these have been omitted from the DDD Plus cohort to avoid subject duplication. Participants in these studies were selected for structural CHD (excluding prematurity-associated patent ductus arteriosus). Patients with known chromosome abnormalities were the only patients excluded from both cohorts. Controls included 1,789 previously analyzed families that included one offspring with autism, one unaffected sibling and unaffected parents; only data from the unaffected siblings and parents were analyzed as controls in this study^40^. Two hundred twenty nine previously published NDD genes were individually annotated with respect to connectome and chromatin modifier status by two independent reviewers (Supplementary Table S2)^47^. PubMed search terms included connectome, neural connectivity, neural network, neuron, neurogenesis, axon, growth cone, dendrite, synapse, synaptogenesis, oligodendroglia, myelinogenesis, chromatin modifier, chromatin and methylation, acetylation and/or ubiquitination. Connectome genes represent a subset of the NDD genes; of the 229 NDD genes (Fig. 1), 159 fulfilled our definition for contribution to the developing connectome, including 30 genes that are also chromatin modifiers.

### Analysis of DNVs of NDD genes in CHD cases and controls

For all analyses, we first report data for the DDD Plus Study (N = 1039) and the PCGC study (N = 2645) independently followed by those for the combined CHD cohort (N = 3684).

As shown in Table 1, we identified the frequency of DNVs in the 229 NDD genes occurring in both the individual CHD populations and the combined CHD cohort, and compared to those found in the control group using Fisher’s exact tests. Results were summarized using odds ratios and 95% Confidence Intervals (95% CI). Single nucleotide variants and small indels were classified into distinct categories: protein truncating (PT, i.e., nonsense, frameshift, splice-site variants), missense variants (MIS; including small in-frame insertions or deletions), and synonymous variants. All missense variants predicted by MetaSVM as deleterious were classified as D-mis variants^40^. Protein damaging variants refer PT or D-mis DNVs.

**Table 1:** *De novo* variants of 229 NDD genes identified from CHD individuals of DDD Plus Study and PCGC cohorts.

Variant Category	DDD Plus Study-Sifrim 2016 (N = 1039)				PCGC-Jin 2017 (N = 2645)				Combined CHD (N = 3684)				Controls (N = 1789)
	Allele Count	OR	95% CI	p-value	Allele Count	OR	95% CI	p-value	Allele Count	OR	95% CI	p-value	Allele Count
Total	94	2.80	2.00, 3.93	1.50E-09	163	1.79	1.32, 2.44	1.52E-04	257	2.07	1.54, 2.77	2.96E-07	57
PT	44	10.48	3.76, 18.31	1.33E-12	62	5.51	2.52, 12.07	2.60E-07	106	6.87	3.19, 14.78	1.72E-10	7
Missense	44	1.90	1.02, 2.38	0.0054	75	1.21	0.82, 1.80	0.38	119	1.40	0.97, 2.02	0.079	38
D-mis	23	3.13	1.19, 4.72	0.0013	33	1.69	0.87, 3.28	0.12	56	2.08	1.11, 3.90	0.017	12
PT or D-mis	67	5.97	2.79, 7.67	4.81E-14	95	3.13	1.90, 5.14	7.10E-07	162	3.89	2.41, 6.28	7.80E-11	19
Non D-mis	21	1.31	0.68, 2.01	0.37	42	0.99	0.60, 1.62	1.0	63	1.08	0.68, 1.70	0.82	26
SYN	6	0.80	0.25, 1.61	0.81	26	1.33	0.67, 2.64	0.50	32	1.18	0.61, 2.30	0.74	12

Compared to the controls, although there was a higher frequency of DNVs in the 229 NDD genes in both the DDD Plus Study and the PCGC cohort (OR = 2.80, 95%CI:2.00–3.93, Fisher’s exact test P = 1.50E-09 and OR = 1.79, 95%CI:1.32–2.44, Fisher’s exact test P = 1.52E-04, respectively), this excess was largely attributable to DNVs in PT (OR = 10.48, 95%CI:3.76–18.31, Fisher’s exact test P = 1.33E-12 (DDD Plus); OR = 5.51, 95%CI:2.52–12.07, Fisher’s exact test P = 2.60E-07 (PCGC)) or protein damaging DNVs (OR = 5.97, 95%:2.79–7.67, Fisher’s exact test P = 4.81E-14 (DDD Plus); OR = 3.13, 95%CI:1.90–5.14, Fisher’s exact test P = 7.10E-07 (PCGC)), as shown in Table 1. In contrast, we did not see any significant changes in synonymous or non-D-mis DNVs in the NDD genes between the CHD cases and controls in either of the separate primary studies or the analysis with combined data. Among the 3684 CHD subjects, 162 were carriers of PT or D-mis DNVs in NDD genes, which accounted for 4.3% of individuals in this cohort, including 3 individuals with 2 PT or D-mis variants in two different genes. In addition, the enrichment of gene burden of PT DNV*s* in the NDD genes was observed when compared to the whole exomes in the DDD Plus or PCGC primary study cohorts (OR = 10.48, 95%CI:3.76–18.31 or OR = 5.51, 95%CI:2.52–12.07 vs OR = 1.89, 95%CI:1.49–2.39 or OR = 1.60, 95%CI:1.31–1.95, respectively; Table 1 and Supplementary Table S1). The excess of protein damaging DNVs were aggregated in genes contributing to both heart development and NDD in this analysis, further confirming previous data from the PCGC study^40^.

### Analysis of DNVs of connectome genes in CHD cases and controls

Of the 229 NDD genes (Fig. 1), 159 fulfilled our definition for contribution to the developing connectome (i.e., genes contributing to neurogenesis, axonal migration, dendritic development, myelinogenesis and/or synaptogenesis). We next compared the DNVs in these 159 connectome genes occurring in the CHD population to those found in the control group, using the same statistical methodology as above, as shown in Table 2. We identified 134 PT or D-mis DNVs derived from 47 connectome genes. Similar to the NDD data above, there were more protein damaging variants in connectome genes in either the DDD Plus Study (OR = 8.14, 95%CI:4.35–15.2, Fisher’s exact test P = 1.38E-14) or the PCGC cohort (OR = 3.95, 95%CI:2.14–7.28, Fisher’s exact test P = 5.99E-07) compared to controls. The odd ratios became even more striking for DDD Plus Study (OR = 14.93, 95%CI:5.30–42.0) and PCGC Study (OR = 7.13, 95%CI:2.56–19.8), respectively, if only PT DNVs were included.

**Table 2:** *De novo* variants of connectome genes identified from the two CHD cohorts.

Variant Category	DDD Plus Study-Sifrim 2016 (N = 1039)				PCGC-Jin 2017 (N = 2645)				Combined CHD (N = 3684)				Controls (N = 1789)
	Allele Count	OR	95% CI	p-value	Allele Count	OR	95% CI	p-value	Allele Count	OR	95% CI	p-value	Allele Count
Total	83	3.27	2.25, 4.77	2.25E-10	123	1.78	1.25, 2.54	1.08E-03	206	2.18	1.56, 3.05	1.07E-06	43
PT	36	14.93	5.30, 42.0	1.06E-11	46	7.13	2.56, 19.8	2.41E-06	82	9.25	3.39, 25.3	2.13E-09	4
Missense	42	2.56	1.57, 4.18	1.42E-04	55	1.25	0.79, 1.99	0.36	97	1.61	1.04, 2.47	0.03	27
D-mis	22	4.49	1.99, 10.1	1.07E-04	30	2.31	1.05, 5.04	0.042	52	2.91	1.11, 3.90	2.79E-03	8
PT or D-mis	58	8.14	4.35, 15.2	1.38E-14	76	3.95	2.14, 7.28	5.99E-07	134	5.08	2.81, 9.20	6.30E-11	12
Non D-mis	20	1.71	0.91, 3.21	0.10	25	0.80	0.44, 1.46	0.53	45	1.05	0.61, 1.80	1.0	19
SYN	5	0.67	0.24, 1.90	0.62	22	1.12	0.54, 2.27	0.86	27	1.00	0.51, 1.97	1.0	12

Similarly when the cohorts were combined, CHD subjects had a higher frequency of protein damaging DNVs among connectome genes than control subjects (OR = 5.08, 95%CI:2.81–9.20, Fisher’s exact test P = 6.30E-11, in which the excess was largely contributed by PT DNVs (OR = 9.25, 95%CI:3.39–25.3, Fisher’s exact test P = 2.13E-09 Table 2). Cases also had a higher fraction of total DNVs contributing to the connectome when compared to control subjects (OR = 2.18, 95% CI:1.56–3.05, Fisher’s exact test P = 1.07E-06). In contrast, we did not see any significant differences for synonymous or non-Dmis DNVs in the connectome genes between the CHD cases and controls.

### Analysis of Chromatin Modifier DNVs in NDD genes in CHD cases and controls

Chromatin modifiers have been reported to play a significant role in neural development, and we identified 60 DNVs from 18 NDD genes described as chromatin modifiers. As shown in Table 3, there were more PT DNVs in chromatin modifier genes in the DDD Plus Study (OR = 15.53, 95%CI:3.61–66.8, Fisher’s exact test P = 9.53E-07) or the PCGC cohort (OR = 7.71, 95%CI:1.82–32.6, Fisher’s exact test P = 5.10E-04), compared to controls. There were also more protein damaging DNVs in chromatin modifier genes in the DDD Plus Study (OR = 6.33, 95%CI:2.75–14.6, Fisher’s exact test P = 1.13E-06) and the PCGC subjects (OR = 2.91, 95%CI:1.28–6.58, Fisher’s exact test P = 8.04E-03), respectively.

**Table 3:** *De novo* variants of chromatin modifier genes identified from the two CHD cohorts.

Variant Category	DDD Plus Study-Sifrim 2016 (N = 1039)				PCGC-Jin 2017 (N = 2645)				Combined CHD (N = 3684)				Controls (N = 1789)
	Allele Count	OR	95% CI	p-value	Allele Count	OR	95% CI	p-value	Allele Count	OR	95% CI	p-value	Allele Count
Total	32	3.28	2.79, 6.01	7.15E-05	49	1.89	1.07, 3.33	0.028	81	2.27	1.32, 3.89	0.0017	16
PT	19	15.53	3.61, 66.8	9.53E-07	25	7.71	1.82, 32.6	5.10E-04	44	9.85	2.38, 40.7	1.55E-05	2
Missense	12	1.94	0.84, 4.51	0.13	17	1.04	0.47, 2.28	1.0	29	1.29	0.63, 2.65	0.60	10
D-mis	8	2.59	0.84, 7.92	0.094	8	0.98	0.32, 3.00	1.0	16	1.42	0.52, 3.88	0.64	5
PT or D-mis	27	6.33	2.75, 14.6	1.13E-06	33	2.91	1.28, 6.58	8.04E-03	60	3.84	1.75, 8.42	1.44E-04	7
Non D-mis	4	1.29	0.34, 4.81	0.74	9	1.10	0.37, 3.29	1.0	13	1.15	0.41, 3.24	1.0	5
SYN	0	0.18	0.01, 3.32	0.30	7	1.07	0.31, 3.66	1.0	7	0.77	0.23, 2.65	0.74	4

Comparison of the number of DNVs in chromatin modifier genes occurring in the NDD genes in the combined cohort of CHD cases and controls demonstrated an excess of protein damaging DNVs (OR = 3.84, 95%CI: 1.75–8.42, Fisher’s exact test P = 1.44E-04) in CHD subjects with greater contribution from PT DNVs (OR = 9.85, 95%CI:2.38–40.7, Fisher’s exact test P = 1.55E-05; Table 3). Of note, we did not observe any significant differences between the CHD cases and controls with respect to synonymous or non-Dmis DNVs of chromatin modifier genes.

### Identification of 12 NDD candidate genes with higher DNV burden in the CHD population

To identify a subset of NDD genes in which damaging DNVs are over-represented in the CHD cohorts, we implemented a one-tailed binomial test to quantify the enrichment of protein damaging DNVs in 229 NDD genes in only CHD cases from the DDD Plus study and PCGC study. This method calculated the exact probability of the observed data under a binomial distribution with each gene mutation rate as a specified probability parameter^53,54^. The observed top 38 enriched NDD genes with protein damaging DNVs more than expected (ranked by one-tailed binomial test p-value, P < 0.05) are shown in Table 4.

**Table 4:** Top NDD Genes (adjusted by mutation rate, one-tailed binomial test p < 0.05) with *de novo* PT or D-mis variants from the two CHD cohorts.

Gene	Chromatin Modifiers	Connectome	Mutation Rate	Size	DDD-Plus	PCGC	Observed	Expected	P-value
PTPN11	−	+	2.58E-05	1801	7	9	16	0.80	1.54E-34
CHD7	+	+	1.19E-04	8909	3	14	17	3.96	7.56E-26
CHD4	+	+	8.01E-05	5678	3	3	6	2.52	2.29E-08
KMT2A	+	+	1.48E-04	11810	5	2	7	5.25	3.49E-08
NOTCH1	−	+	1.59E-04	7699	1	5	6	3.42	1.21E-06
ADNP	−	+	3.92E-05	3299	4	0	4	1.47	1.59E-06
SMAD2	−	+	1.71E-05	1410	0	3	3	0.63	6.84E-06
KDM5B	+	+	6.13E-05	4662	1	3	4	2.07	9.19E-06
NSD2	+	+	2.56E-05	4192	1	2	3	1.86	2.27E-05
FOXP1	−	+	3.02E-05	2216	3	0	3	0.99	3.69E-05
MED13L	−	+	8.79E-05	6615	3	1	4	2.94	3.72E-05
DYRK1A	−	+	3.12E-05	2387	3	0	3	1.06	4.08E-05
POGZ	−	+	5.66E-05	4179	0	3	3	1.86	2.35E-04
PTEN	−	+	1.40E-05	1326	0	2	2	0.59	3.97E-04
KAT6A	+	+	8.14E-05	5995	2	1	3	2.66	6.73E-04
ARID1B	+	+	9.02E-05	6412	2	1	3	2.85	9.03E-04
SMAD4	−	+	2.14E-05	1670	1	1	2	0.74	9.28E-04
EP300	+	+	9.32E-05	7214	2	1	3	3.21	9.92E-04
WAC	+	−	2.42E-05	1958	1	1	2	0.87	1.18E-03
FRYL	−	−	1.07E-04	9100	1	2	3	4.04	1.45E-03
NAA15	−	+	2.84E-05	2621	0	2	2	1.17	1.61E-03
DDX3X	−	+	2.90E-05	2006	1	1	2	0.89	1.68E-03
CUL3	−	−	2.98E-05	2323	1	1	2	1.03	1.77E-03
CTNNB1	−	+	2.99E-05	2332	0	2	2	1.04	1.78E-03
ANKRD11	+	+	1.59E-04	7964	3	0	3	3.54	4.44E-03
LZTR1	+	−	4.81E-05	2544	0	2	2	1.13	4.50E-03
SETD5	+	−	5.43E-05	4329	1	1	2	1.92	5.69E-03
ASH1L	+	+	1.07E-04	8868	1	1	2	3.94	2.05E-02
NAT8L	−	+	1.06E-05	912	0	1	1	0.41	2.13E-02
TECTA	−	−	1.10E-04	6255	1	1	2	2.78	2.18E-02
ITPR1	−	−	1.18E-04	8337	2	0	2	3.71	2.45E-02
NTM	−	+	1.77E-05	1176	0	1	1	0.52	3.55E-02
PSMD12	−	−	1.82E-05	1382	0	1	1	0.61	3.64E-02
PCOLCE	−	−	2.19E-05	1359	0	1	1	0.60	4.37E-02
EP400	+	−	1.63E-04	9360	1	1	2	4.16	4.45E-02
ANK3	−	+	1.67E-04	13243	0	2	2	5.89	4.63E-02
CCSER1	−	−	2.35E-05	2705	0	1	1	1.20	4.68E-02
KMT2C	+	+	1.74E-04	14569	0	2	2	6.48	4.97E-02

Six genes (*PTPN11, CHD7, CHD4, KMT2A, NOTCH1, ADNP*) reached genome wide significance (P ≤ 2.5E-06, Bonferroni correction), and the top two genes were *PTPN11* (one-tailed binomial test P ≤ 1.54E-34) and *CHD7* (one-tailed binomial test P ≤ 7.56E-26), which are associated with Noonan syndrome (OMIM:163950) and CHARGE syndrome (OMIM:214800). The pathogenic missense variants encoding the same p.Asn308 residue of PTPN11 ([hg19], chr12:112915523 A > G, p.N308D; chr12:112915524 A > G, p.N308S; or chr12:112915524 A > C, p.N308T) were the most recurrent amino acid substitutions observed in 7 out of 16 individuals, whereas in CHD7, all were unique singleton variants - with 76.5% (13/17) as PT DNVs. These observations are consistent with the most likely pathogenic mechanisms underlying these two genetic conditions^55–57^.

**Figure 2:**
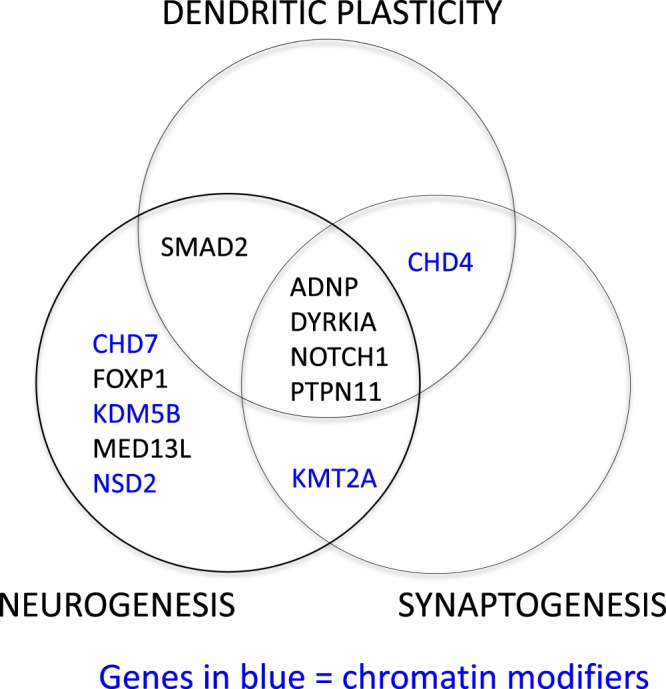
Functions of the top 12 over-represented genes with damaging DNVs. Review of the published literature for the identified top 12 enriched genes achieving statistical significance after correction for multiple comparisons revealed that 11 contributed to neurogenesis and one, *CHD4*, contributed to dendritic plasticity and synaptogenesis. Of the 11 contributing to neurogenesis, four genes, *ADNP, DYRKIA, NOTCH1* and *PTPN11*, also contributed to dendritic plasticity and synaptogenesis. One, *SMAD2*, also subserved dendritic plasticity, and a single gene, KMT2A contributed to both neurogenesis and synaptogenesis. Five of the top genes were chromatin modifiers.

Review of the top twelve NDD genes (*PTPN11, CHD7, CHD4, KMT2A, NOTCH1, ADNP, SMAD2, KDM5B, NSD2, FOXP1, MED13L* and *DYRK1A*) with higher burden for the protein damaging DNVs (one-tailed binomial test P ≤ 2.18E-04, statistical significance after Bonferroni correction) showed that all 12 contributed to the connectome, and 5 of them belonged to chromatin modifiers. This enrichment could also be observed in the gene numbers for the two categories (connectome Fisher’s exact test P = 0.02, and chromatin modifiers Fisher’s exact test P = 0.04). Finally, as shown in Table 5, review of the available literature showed that 11 of these genes contribute to neurogenesis; 6 play a role in dendrite formation, and 6 contribute to synaptogenesis. As shown in Fig. 2 there was a sizeable overlap among the roles of these genes, while *ADNP*^58–61^, *DYRKIA*^62,63^, *NOTCH1*^64–66^ and *PTPN11*^67–70^ are reported to contribute to neurogenesis, dendrite formation and synaptogenesis.

**Table 5:** Reported functions of the top 12 NDD genes (one-tailed binomial test p-value cutoff <2.18E-04, statistical significance after Bonferroni correction).

Gene	Chromatin Modifier	Cerebellum	Connectome	Neurogenesis	Dendrites	Synapse	Syndrome
ADNP	−	−	+	+	+	+	Helsmoortel-van der AA
CHD4	+	+	+	−	+	+	Sifrim-Hitz-Weiss Syndrome
CHD7	+	+	+	+	−	−	CHARGE Syndrome
DYRK1A	−	−	+	+	+	+	Down Syndrome
FOXP1	−	+	+	+	−	−	FOXP1-related ID Syndrome
KDM5B	+	−	+	+	−	−	ID syndrome
KMT2A	+	−	+	+	-	+	Wiedemann-Steiner Syndrome
MED13L	−	-	+	+	−	−	ID, corpus callosum changes
NOTCH1	-	+	+	+	+	+	Adams Oliver Syndrome
NSD2	+	−	+	+	−	−	Wolf Hirschorn Syndrome
PTPN11	−	+	+	+	+	+	Noonan Syndrome
SMAD2	−	+	+	+	+	−	ID, dysmorphic features, FTT

## Discussion

Converging data suggest that genes that contribute to structural heart development also contribute to the connectome, and we identified 12 candidate genes supporting this hypothesis. These candidates, including *ADNP, CHD4, CHD7, DYRK1A, FOXP1, KDM5B, KMT2A, MED13L, NOTCH1, PTPN11, SMAD2* and *NSD2*, contribute to a broad array of both well-established and more recently identified NDD syndromes. Eleven out of 12 sub-serve neurogenesis; 5 are chromatin modifiers and 2 are members of the NOTCH pathway (Table 5). Finally, the contribution of the cerebellum to cognition and behavior has recently been reported^71,72^, and 6 of the twelve identified genes are known to contribute to cerebellar development.

Three of these genes are associated with well-known pediatric syndromes.

- Mutations in the RAS-MAPK pathway are associated with NDD, while Noonan syndrome is the most common clinical RASopathy. In preclinical models, *PTPN11*, the gene responsible for 50% of cases of Noonan Syndrome, regulates neurogenesis and is required for neuronal process extension^67,68^. In addition, *PTPN11* differentially regulates expression of post-synaptic receptors and contributes to synaptic homeostasis, while variants in this gene alter surface expression of both AMPA and NMDA receptors during development^69,70^.
- DYRK1A contributes to cognitive disability in Down syndrome. While trisomies were omitted from our -analysis, we noted 4 subjects with *de novo DYRK1A* variants. Three had PT DNVs, and a fourth was noted to have a deleterious missense DNV. Haplo-insufficiency of DYRK1A results in ID and CHD^73^, and preclinical studies demonstrate decreased striatal dopamine levels, reduced number of dopamine neurons in the substantia nigra pars compacta and altered behavioral responses to dopaminergic agents, suggesting that haplo-insufficiency of DYRK1A alters the connectome^62^. In contrast, over-expression of DYRK1A increases the population of GABA inter-neurons and alters the excitatory or inhibitory synapse balance in developing brain^63^.
- Mutations in the ATP-dependent chromatin remodeler *CHD7* are responsible for CHARGE syndrome (**C**oloboma, **H**eart defects, **A**tresia choanae, **R**etarded growth and development and **G**enital and **E**ar abnormalities). Variants in *CHD7* have been described in subjects with both autism and ID, and many CHARGE patients show hypoplasia of the cerebellum. CHD7 is present in both neuronal precursors and stem cells, with genetic inactivation of CHD7 in cerebellar granule neuron progenitors leading to cerebellar hypoplasia in mice due to impaired granule neuron differentiation, apoptosis and abnormal Purkinje cell migration^50^.

Two additional candidates, *CHD4* and *SMAD2*, also play important roles in cerebellar development, CHD and intellectual disability^74^.

- In preclinical studies, knock-out of Chd4 impairs dendritic pruning in developing cerebellar granule neurons and impedes the establishment of granule neuron parallel fiber or Purkinje cell synapses in the rodent cerebellar cortex^45,75,76^. Recent publications describe an autosomal dominant ID syndrome attributable to variants in *CHD4*; affected patients also show cardiac, skeletal and urogenital malformations^48,74^.
- Loss of function *SMAD2* variants cause a wide spectrum of autosomal dominant aortic and arterial aneurysmal disease, and a recent report describes two patients with these variants who have complex CHD and NDD. Preclinical studies show delayed migration and maturation of granule cells and retardation of dendritic arborization of Purkinje cells, suggesting that Smad2 plays a key role in cerebellar connectivity^76,77^.

Language disability is common among children with CHD, and subjects with *FOXP1*, *MED13L, NOTCH1* and *ADNP* variants show ID and specific language impairment, with or without ASD^64,78,79^.

- FOXP1 is expressed in neural stem cells, and modulation of FOXP1 expression influences neuronal differentiation. In a preclinical model of cortical development, *FOXP1*-knockdown *in utero* reduced both neural stem cell differentiation and migration. Furthermore, FOXP1 repressed expression of Notch pathway genes, resulting in inhibition of Notch signaling^80^.
- NOTCH1, responsible for Adams Oliver Syndrome, is required for neuronal differentiation, dendrite development and synaptic plasticity in developing brain^64–66^.
- Similarly, subjects with haplo-insufficiently of MED13L show ID and severe speech delay; congenital heart defects are found in 20–50% of patients^79,81^. In preclinical studies, haploinsufficiency of MED13L shows defects in both neuronal migration and differentiation^82,83^.
- *ADNP* variants are reported in children with autism and ID who carry a diagnosis of Helsmoortel-Van der Aa syndrome^84^. In preclinical studies, ADNP deficiency decreases neurogenesis, reduces dendritic spine density, impairs neurite outgrowth and alters synaptic gene expression^58–61^.

Finally, histone lysine methyltransferases (KMTs) and demethylases (KDMs) are posited to regulate gene regulation, while variants causing haplo-insufficiency of KMTs and KDMs are common in patients with NDD^85^.

- Dominant DNVs in *KMT2A* have been reported in individuals with Wiedemann-Steiner syndrome, a developmental disorder with ID and cardiac anomalies; KMT2A peaks in expression in human fetal brains and is reported to be essential for both neurogenesis and synaptic plasticity^86–88^.
- Haploinsufficiency of NSD2, a histone lysine methyltransferase, is associated with all known cases of Wolf-Hirschhorn Syndrome^89^. While little is known about the neurobiology of *NSD2* variants, suppression of the functional homolog of *NSD2* in zebrafish affects early embryogenesis, including incomplete neuron formation and endbrain or cerebellar volume changes, which are also observed in Wolf-Hirschhorn patients and Nsd2-deficient mice^89–91^.
- KDM5B, a histone lysine demethylase, negatively regulates neurogenesis, represses Reln expression in neural stem cells in the adult subventricular zone and has been reported to cause ID^92^. Of note, a recent study reports a single patient with a *KDM5B* variant, ID and an atrial septal defect^85^.

In addition to their recognized NDDs, infants and children with CHD are at high risk for abnormal MRI studies of the brain, and recent data suggest a correlation of behavior with alterations in the connectome. To better explore these findings, prior studies have addressed either the impact of targeted CHD variants on brain development and neurodevelopmental outcome^93^, or identified genes that are both highly expressed in the developing heart and contribute to NDD or brain development. However, none have provided analyses of large cohorts with MRI measures of neural connectivity. In addition, the contribution of DNVs in brain or connectome development associated with fetal MRI studies, prior to hypoxemia, are largely lacking^94^.

Genetic testing is an important component of the evaluation for neonates with CHD as it may both impact strategies for clinical care and provide long-term outcome information. Diagnostic genetic variants are detected in 11.1% of fetuses with cardiac anomalies^95^. In addition, the incidence of extra-cardiac malformations patients with CHD has been reported to range from 10–26%^96^, and those CHD patients with extra-cardiac malformations are more likely to harbor pathogenic DNVs than those with only CHD^48,97^. In addition, significant developmental delay defined as a cognitive, language or motor score <70 has been reported to occur in almost 75% of children with a known genetic syndrome, compared to 33% of children with single-ventricle non-syndromic CHD and 20% of those with bi-ventricular non-syndromic CHD^98^. These data demonstrate that while syndromic cases have a higher incidence of developmental delay, children with non-syndromic CHD are also affected by this lifelong disability. The goal of our study is to suggest that these disabilities are not simply due to hypoxia but may indeed have a genetic origin.

The strengths of this study include the analysis of multiple previously published large CHD data sets and the independently reported NDD genes. The weaknesses include the lack of MR connectome imaging and limited phenotyping data for the study subjects. In addition, paternal age, which is a risk factor for DNVs, was not available for our analysis^99,100^. Although all the variants we examined are *de novo* variants and all control subjects were reported to have unremarkable clinical presentations, our controls were first-degree unaffected siblings of subjects with ASD. Finally, although we excluded subjects with aneuploidies, genes contributing to Noonan Syndrome, CHARGE Syndrome and Down Syndrome are among our significant candidates, suggesting a possible bias to syndromic intellectual disability. Nonetheless, after excluding these subjects from our analyses, our major findings persisted (Supplementary Table S3).

As the growing population of children with CHD becomes adolescents and these adolescents transition to adult cardiology care, the impact of NDD on this population does not wane^28,101,102^. Recent data demonstrate a significant prevalence and impact of neurocognitive deficits among adults with CHD; adult CHD subjects have lower academic levels and higher unemployment rates compared to reference populations. Furthermore, adult CHD patients are more likely than typical young adults to suffer from depression. [For review of adult CHD neurocognitive or behavioral deficits, please see^103^]. Establishing the determinants of neuro-behavior in those with CHD will permit both prognostication and targets for intervention, and future work should link genes contributing to cardiac development to the functional connectome.

## Methods

To test the hypothesis that variants contributing to NDD are more common in subjects with CHD than in controls, we performed a secondary analysis of primary data from previously published cohorts of subjects with CHD and genomic data from those with NDD. High confidence DNVs (pp_dnm ≥ 0.9) from Sifrim *et al*. were included^48^, and DNVs from Jin *et al*. were qualified using the previously published filtering criteria followed by examination using *in silico* visualization^40^. We compared the frequency of DNVs in the NDD genes occurring in the CHD population to those found in the control group using Fisher’s exact tests. Results were summarized using odds ratios and 95% Confidence Intervals (95%CI)^104^. To test for over-representation of a gene set among cases, a one-tailed binomial test was conducted by comparing the observed number of variants to the expected count as previously reported^40^. Bonferroni correction was applied for multiple comparisons^105^. Analyses were performed using Excel, Microsoft Office 365, and online software MEDCALC (https://www.medcalc.org/calc/odds_ratio.php), Fisher’s Exact test (https://www.langsrud.com/stat/fisher.htm).

### Subject population

CHD subjects with genomic data were ascertained from recent publications. Evaluation of available subjects from the multi-center/multi-study cohort reported by Deciphering Developmental Disorders Project (“DDD Plus Study”, N = 1039 cases)^48^ and a recent publication from the Pediatric Cardiac Genome Consortium (PCGC) study^40^ (N = 2645 cases) yielded 3684 unique cases of structural CHD (excluding prematurity-associated patent ductus arteriosus). Controls included 1,789 previously analyzed unaffected siblings of autism probands and their unaffected parents^40^.

Analysis of the overall *de novo* variants (DNVs) identified from whole exomes in the DDD Plus Study, PCGC Study and controls (Supplementary Table S1) demonstrated that the mean DNVs per individual are 1.09, 1.13, and 1.02 respectively. Compared to control subjects, both DDD Plus subjects and PCGC subjects had a higher prevalence of protein truncating (PT) variants (OR = 1.89, 95%CI:1.49–2.39, Fisher’s exact test P = 1.53E-07, DDD Plus Study; and OR = 1.60, 95%CI: 1.31–1.95, Fisher’s exact test P = 2.25E-06, PCGC Study). In addition, DDD Plus and PCGC subjects had similar prevalence of both *de novo* missense and synonymous variants when compared to controls, suggesting similarity of the data sets.

### Target genes

The 229 target NDD disease-risk genes (ASD, DD and ID) were selected based on recently published sequencing studies^47,106,107^. Genes contributing to the connectome were defined as those necessary for the development, growth and maintenance of neural networks in developing brain^49–51^, while chromatin modifiers were defined as variants that alter the assembly and compaction of chromatin^52^. Each of the 229 candidate genes was individually annotated using PubMed search terms including connectome, neural connectivity, neuron, neurogenesis, axon, growth cone, dendrite, synapse, synaptogenesis, oligodendroglia, myelinogenesis, chromatin modifier, chromatin and methylation, acetylation and/or ubiquitination (Supplementary Table 2). Functions and murine phenotypes of these genes (Mouse Genome Informatics, http://www.informatics.jax.org/) are also shown in Supplementary Table 2. Gene assignments are effective 6–30–2018, and a diagram demonstrating the inter-relationship of NDD, connectome and chromatin modifier genes for the 229 target genes is shown in Fig. 1.

### Ethical approval and informed consent

The Yale University IRB does not require approval for meta-analyses involving de-identified data.

## Supplementary information

Supplementary Information.

## Data Availability

The datasets generated and/or analyzed during the current study are available from the corresponding author on request.
