# Supplementary material for: De novo damaging variants associated with congenital heart diseases contribute to the connectome
Source: Sci Rep. 2020 Apr 27;10:7046. doi: 10.1038/s41598-020-63928-2 (PMC7184603; doi:10.1038/s41598-020-63928-2)
Supplement: Supplementary file 1 — Supplementary Information. [file 41598_2020_63928_MOESM1_ESM.docx]

Supplementary Information

***De novo* damaging variants associated with congenital heart diseases**

**contribute to the connectome**

**Weizhen Ji^1^, Dina Ferdman^1^, Joshua Copel^1, 2^, Dustin Scheinost^3^, Veronika Shabanova^1^, Martina Brueckner^1, 4, 5,^** ^+^**, Mustafa K Khokha^1, 4,^** ^+^**, and Laura R Ment^1, 6,^** ^+,^ **^*^**

**Supplementary Table S1**. WES *de novo* variants identified from CHD subjects from DDD Plus Study and PCGC cohorts.

|  |  | **DDD Plus Study-Sifrim 2016**  **(N=1039)** | | | | | **PCGC-Jin 2017**  **(N=2645)** | | | | | **Controls**  **(N=1789)** | |  |
| --- | --- | --- | --- | --- | --- | --- | --- | --- | --- | --- | --- | --- | --- | --- |
| Variant  Category | Allele Count | | Alleles per subject | OR | 95% CI | p-value | Allele Count | Alleles per subject | OR | 95% CI | p-value | Allele Count | Alleles per subject | |
| Total | 1137 | | 1.09 | - | - | - | 2990 | 1.13 | - | - | - | 1830 | 1.02 | |
| PT | 164 | | 0.16 | 1.89 | 1.49, 2.39 | 1.53E-07 | 374 | 0.14 | 1.60 | 1.31, 1.95 | 2.25E-06 | 150 | 0.08 | |
| Missense | 746 | | 0.72 | 1.01 | 0.86, 1.18 | 0.89 | 1915 | 0.72 | 0.94 | 0.84, 1.07 | 0.36 | 1196 | 0.67 | |
| SYN | 227 | | 0.22 | 0.69 | 0.58, 0.83 | 0.0001 | 701 | 0.27 | 0.85 | 0.74, 0.97 | 0.02 | 484 | 0.27 | |

**Supplementary Table S2**. Functions of the 229 NDD genes.

| Gene | Gene Name | Chromatin Modifier | Connectome | Neurogenesis | Axon  /Dendrite | Synaptogenesis | Syndrome: Phenotype [OMIM#] | MGI Phenotype | |
| --- | --- | --- | --- | --- | --- | --- | --- | --- | --- |
| *ABI2* | Abl interactor 2 | - | + | - | + | + | None | |  |
| *ADCY5* | Adenylate cyclase 5 | - | + | - | - | + | Dyskinesia, familial, with facial myokymia [MIM: [606703](https://www.omim.org/entry/606703) ] | | Motor dysfunction |
| *ADCY9* | Adenylate cyclase 9 | - | - | - | - | - | None | | Bradycardia and defects in left ventricular diastolic function. An increased igg1 response to ovalbumin challenge |
| ADGRB1 | Adhesion g protein-coupled receptor b1 | - | + | - | - | + | None | |  |
| *ADGRL2* | Adhesion G protein-coupled receptor L2 | - | - | - | - | - | None | | Homozygous die prenatally. Heterozygous mice exhibit decreased locomotor activity in an open field test. |
| *ADNP* | Activity dependent neuroprotector homeobox | - | + | + | + | + | Helsmoortel-Van der Aa syndrome [MIM: [615873](https://www.omim.org/entry/615873) ] | | Developmental defects including the failure of the cranial neural tube to close lead to embryonic death between E8.5 and E9. |
| *AGAP2* | ArfGAP with GTPase domain, ankyrin repeat and PH domain 2 | - | + | - | - | + | None | | Impaired lactation due to abnormal mammary gland growth during lactation, failure of insulin-suppressed gluconeogenesis, and hyperglycemia. |
| *AGO4* | Argonaute 4, risc catalytic component | - | - | - | - | - | None | | Oligozoospermia, decreased testis weight, premature entry into meiosis and disruption of sex body formation. Both males and females are fertile. |
| *AHNAK* | AHNAK nucleoprotein | - | + | + | - | + | None | | Decreased T cell proliferation and increased susceptibility to parasitic infection |
| *AK8* | Adenylate kinase 8 | - | - | - | - | - | None | | Mild hydrocephalus, dilation of the lateral brain ventricles and reduced size of the hippocampus |
| *ALDH5A1* | Aldehyde dehydrogenase 5 family member A1 | - | + | - | - | + | Succinic semialdehyde dehydrogenase deficiency [MIM: [271980](https://www.omim.org/entry/271980) ] | | Reduced body weight, ataxia, seizures, gliosis of the hippocampus, and early death |
| *ANK2* | Ankyrin 2 | - | + | - | + | + | Long QT syndrome 4; Cardiac arrhythmia, ankyrin-B-related [MIM: [600919](https://www.omim.org/entry/600919) ] | | Died by postnatal day 8, although some animals survive to P20. Display reduced body size, impaired balance and locomotion, brain structure dysmorphologies, abnormal lens, and optic nerve degeneration. |
| *ANK3* | Ankyrin 3 | - | + | - | + | + | Mental retardation, autosomal recessive, 37 [MIM: [615493](https://www.omim.org/entry/615493) ] | | Progressive ataxia, tremors, and a substantially reduced cerebellum deficient in Purkinje cells. Mutants are poor breeders and die by 4-6 months. |
| *ANKRD11* | Ankyrin repeat domain 11 | + | + | + | - | - | KBG syndrome [MIM: [148050](https://www.omim.org/entry/148050) ] | | Mice homozygous for a spontaneous allele die by E9.5, are small and fail to turn. Mice heterozygous for a spontaneous allele exhibit craniofacial abnormalities, decreased weight, osteoporosis and osteopenia. |
| *ANP32A* | Acidic nuclear phosphoprotein 32 family member a | + | + | + | - | - | None | | Viable, fertile, behaviorally normal, and show no defects of the central nervous system. |
| *APH1A* | Aph-1 homolog a, gamma-secretase subunit | - | - | - | - | - | None | | Die by E11, are severely growth retarded by E9.5 and display defects in somite patterning, branchial arch and heart chamber development, vascular morphogenesis of the yolk sac and have distended pericardial sacs. |
| *ARHGAP30* | Rho GTPase activating protein 30 | - | - | - | - | - | None | |  |
| *ARHGAP32* | Rho GTPase activating protein 32 | - | + | - | + | - | None | | Fertile but display abnormal neurite growth. |
| *ARHGAP5* | Rho GTPase Activating Protein 5 | - | + | - | + | + | None | | Homozygotes die at birth, are 30% smaller, do not inflate their lungs, and show a small thymus, abnormal adipocyte differentiation and brain defects in the corpus callosum, anterior commissure and lateral ventricles. Mutant MEFs show impaired adipogenesis but undergo myogenesis in response to IGF-1. |
| *ARID1B* | AT-rich interaction domain 1B | + | + | + | + | - | Coffin-Siris syndrome 1 [MIM: [135900](https://www.omim.org/entry/135900) ] | | Mice homozygous for a null allele die prenatally. Heterozygous null mice exhibit increased self-grooming, altered vocalization and response to social novelty, anxiety-like behavior, neuroanatomical anomalies, decreased plasma IGF1 levels, muscle weakness, and growth impairment. |
| *ARIH1* | Ariadne RBR E3 ubiquitin protein ligase 1 | - | - | - | - | - | None | |  |
| *ARVCF* | ARVCF, delta catenin family member | - | + | + | - | - | None | | Abnormal behavior, abnormal gait, cataract, hyperactivity |
| *ARX* | Aristaless related homeobox | - | + | + | - | - | Epileptic encephalopathy, early infantile, 1; Partington syndrome [MIM:300382] | | Males hemizygous for targeted null mutations die prenatally. Male mice hemizygous for various alleles with point mutations or triple repeat expansion exhibit defective gabaergic neuron migration and numbers, seizures, and/or behavioral defects. |
| *ASH1L* | ASH1 like histone lysine methyltransferase | + | + | - | - | + | Mental retardation, autosomal dominant 52 [MIM: [617796](https://www.omim.org/entry/617796) ] | | Homozygotes for a transposon-induced allele are more susceptible to endotoxin shock, sepsis, and autoimmune disease. Homozygotes for a hypomorphic allele show reduced growth and postnatal lethality; surviving adults lack Meibomian glands and show vertebral, reproductive organ, and fertility defects. |
| *ASXL3* | ASXL transcriptional regulator 3 | + | - | - | - | - | Bainbridge-Ropers syndrome [MIM: [615485](https://www.omim.org/entry/615485) ] | |  |
| *CACNA2D3* | Calcium voltage-gated channel auxiliary subunit alpha2delta 3 | - | + | - | - | + | None | | Decreased startle reflex and occasional animals show increased aggression and hyperactivity. |
| *CAPRIN1* | Cell cycle associated protein 1 | - | + | - | - | + | None | | Decreased fetal size, bent posture, cyanosis, respiratory failure, and neonatal lethality with impaired neuronal network development and reduced dendritic localization of sodium potassium ATPase subunit isoform mRNAs. |
| *CASK* | Calcium/calmodulin dependent serine protein kinase | - | + | - | - | + | FG syndrome 4, Mental retardation [MIM: [300422](https://www.omim.org/entry/300422) ] | | Cleft palate and perinatal lethality in hemizygous males and death within 2 weeks in females on a C57BL/6J background. Some female animals on a CD1 background survive to adulthood exhibiting patchy fur, wrinkled skin, a kinked tail and spine, and give birth to small and infrequent litters. Male and female animals on all genetic backgrounds exhibit reduced head size, shortened jaw, and a pointed snout. |
| *CCDC88C* | Coiled-coil domain containing 88C | - | - | - | - | - | Hydrocephalus, congenital, 1 [MIM: [236600](https://www.omim.org/entry/236600) ];  Spinocerebellar ataxia 40 [MIM: [616053](https://www.omim.org/entry/616053) ] | |  |
| CCSER1 | Coiled-coil serine rich protein 1 | - | - | - | - | - | None | |  |
| *CDC42BPB* | CDC42 binding protein kinase beta | - | + | - | + | - | None | | Preweaning lethality, incomplete penetrance |
| *CDC45* | Cell division cycle 45 | - | + | + | - | - | Meier-Gorlin syndrome 7 [MIM: [617063](https://www.omim.org/entry/617063) ] | | Homozygous mutant embryos do not develop after implantation, resulting in embryonic lethality between E4.5-E5.5. Heterozygous animals appear normal and fertile. |
| *CDH10* | Cadherin 10 | - | + | - | - | + | None | | Microphthalmia |
| *CDKL5* | Cyclin dependent kinase like 5 | - | + | + | + | + | Epileptic encephalopathy, early infantile, 2 [MIM: [300672](https://www.omim.org/entry/300672) ] | | Male mice hemizygous for a knock-out allele exhibit hyperactivity, impaired coordination, decreased anxiety-related response, limb grasping, social withdrawal, and impaired nesting, conditioned behavior learning and auditory-evoked event-related potentials. |
| *CHD1* | Chromodomain helicase DNA binding protein 1 | + | + | + | + | + | Pilarowski-Bjornsson syndrome [MIM: [617682](https://www.omim.org/entry/617682) ] | | Complete embryonic lethality associated with arrest of epiblast development due to increased apoptosis and cell cycle defects, abnormal rostral-caudal axis patterning, and failure to gastrulate. |
| *CHD2* | Chromodomain helicase DNA binding protein 2 | + | + | + | - | - | Epileptic encephalopathy, childhood-onset [MIM: [615369](https://www.omim.org/entry/615369) ] | | Early postnatal lethality associated with fetal growth retardation. Heterozygous mutant exhibit postnatal lethality and premature death after weaning associated with growth retardation and multi-organ defects. |
| *CHD4* | Chromodomain helicase DNA binding protein 4 | + | + | - | + | + | Sifrim-Hitz-Weiss syndrome [MIM: [617159](https://www.omim.org/entry/617159) ] | | Embryonic lethality between E3.5 and E4.5, absent blastocoele failure of trophectoderm function and increased apoptosis in blastocysts. |
| *CHD7* | Chromodomain helicase DNA binding protein 7 | + | + | + | - | - | CHARGE syndrome [MIM: [214800](https://www.omim.org/entry/214800) ] | | Heterozygotes for mutations of this gene exhibit a variety of combinations of hyperactivity, circling, head-bobbing, semicircular canal defects, hearing loss, reduced size, and tail-kink. |
| *CHD8* | Chromodomain helicase DNA binding protein 8 | + | + | + | - | - | {Autism, susceptibility to, 18} [MIM: [615032](https://www.omim.org/entry/615032) ] | | Growth retarded starting at E5.5 and exhibit developmental arrest at E6.5. Mutants develop into an egg cylinder but do not form a primitive streak or mesoderm and exhibit increased apoptosis at E7.5. |
| *CHRNA7* | Cholinergic receptor nicotinic alpha 7 subunit | - | + | - | - | + | None | | Lack hippocampal fast nicotinic currents but show nicotine-induced seizures as well as altered anxiety behavior, fertility defects, airway basal cell hyperplasia. And higher tnf synthesis when endotoxemic. Newborns homozygous for a knock-in allele die with increased neuron apoptosis. |
| *COL4A3BP* | Collagen type iv alpha 3 binding protein | - | - | - | - | - | Mental retardation, autosomal dominant 34 [MIM: [616351](https://www.omim.org/entry/616351) ] | | Embryonic lethality during organogenesis with reduced embryo size, impaired heart function, abnormal heart morphology, abnormal mitochondrial morphology and physiology, abnormal endoplasmic reticulum morphology, and decreased cell proliferation. |
| *CREBBP* | CREB binding protein | + | + | + | - | - | Rubinstein-Taybi syndrome 1 [MIM: [180849](https://www.omim.org/entry/180849) ] | | Homozygotes for null or altered alleles die around midgestation with defects in hemopoiesis, blood vessel formation, and neural tube closure. Heterozygotes may exhibit skeletal, cardiac, and hematopoietic defects, retarded growth, and hematologic tumors. |
| *CSMD1* | CUB and Sushi multiple domains 1 | - | + | - | + | - | None | | Normal pre-pulse inhibition, social interaction, sucrose preference and d-amphetamine sensitivity. |
| *CSTF2T* | Cleavage stimulation factor subunit 2 tau variant | - | - | - | - | - | None | | Male mice homozygous for a targeted null allele are infertile due to low sperm counts, significant developmental defects in spermiogenesis, and variable abnormalities in epididymal sperm morphology and motility consistent with oligoasthenoteratozoospermia. Homozygous null females are fertile. |
| *CTNNB1* | Catenin beta 1 | - | + | + | - | + | Neurodevelopmental disorder with spastic diplegia and visual defects [MIM: [615075](https://www.omim.org/entry/615075) ] | | Anterior-posterior axis formation anomalies, but develop to E7. Multiple conditional mutations have shown defects in distinct stem cell types that result in proliferation defects, such as intestinal polyps, brain and spinal cord size anomalies, etc. |
| *CTNND2* | Catenin delta 2 | - | + | - | + | - | ID phenotype (PMID: 25839933) | | Mice homozygous for a reporter allele exhibit abnormal conditioning, spatial learning and coordination behaviors and abnormal long term potentiation. |
| *CTTNBP2* | Cortactin binding protein 2 | - | + | - | + | + | None | |  |
| *CUL3* | Cullin 3 | - | - | - | - | - | Pseudohypoaldosteronism, type IIE [MIM: [614496](https://www.omim.org/entry/614496) ] | | Homozygotes for a targeted null mutation accumulate cyclin E, exhibit abnormal cycling in cells of extraembryonic ectoderm and trophectoderm, reduced size, abnormal gastrulation and trophoblast cells, absence of an amnion, and death by embryonic day 7.5. |
| *CUL7* | Cullin 7 | - | + | - | + | - | 3-M syndrome 1 [MIM: [273750](https://www.omim.org/entry/273750) ] | | During late gestation, homozygous null fetuses display reduced growth associated with abnormal placental development and hemorrhaging due to vascular defects. Mutant mice are born but die shortly after birth, succumbing to respiratory distress. |
| *DCX* | Doublecortin | - | + | + | + | - | Lissencephaly, X-linked [MIM: [300067](https://www.omim.org/entry/300067) ] | | Males hemizygous for a null allele are fertile but show branching and nucleokinesis defects in migrating interneurons. Males hemizygous for a reporter allele show severe postnatal lethality and variable fertility; both female and male mutants display hippocampal dyslamination and behavioral defects. |
| *DDX3X* | DEAD-box helicase 3 x-linked | - | + | - | + | - | Mental retardation, X-linked 102 [MIM: [300958](https://www.omim.org/entry/300958) ] | | Hemizygous males and heterozygous females with a maternally inherited null allele show early embryonic and fetal lethality, respectively. Both males and females show defects in trophoblast giant cells. Females show defects in placental formation. |
| *DHX9* | DExH-box helicase 9 | - | + | + | - | - | None | | Homozygotes die in embryonic stages with massive apoptotic cell death in embryonic ectodermal cells. |
| *DIP2A* | Disco interacting protein 2 homolog A | - | + | - | + | - | None | |  |
| *DLG2* | Discs large MAGUK scaffold protein 2 | - | + | - | - | + | None | | Lower surface expression of NMDA receptor (NMDAR) subunits NR2A and NR2B in dorsal horn neurons and significantly reduced NMDAR-mediated excitatory synaptic currents and NMDAR-dependent persistent inflammatory or nerve injury-induced neuropathic pain. |
| *DLG4* | Discs large MAGUK scaffold protein 4 | - | + | - | - | + | None | | Severely impaired spatial learning, alterations in long-term potentiation and depression, and lack of hyperalgesia responses in a neuropathic pain model. |
| *DLGAP1* | DLG associated protein 1 | - | + | - | - | + | None | | Deficits in sociability. |
| *DNAJC6* | Dnaj heat shock protein family (Hsp40) member C6 | - | + | - | - | + | Parkinson disease 19, juvenile-onset [MIM:608375} | | Postnatal lethality and decreased body weight with homozygotes exhibiting decreased synpatic vesicle recycling. |
| *DOCK8* | Dedicator of cytokinesis 8 | - | - | - | - | - | Hyper-IgE recurrent infection syndrome [MIM:611432] | | Loss of marginal zone B cells, decrease in peritoneal B1 cells and peripheral naive T cells, failure of sustained antibody response after immunization, failure of germinal center persistence, and failure of B cell affinity maturation. |
| *DSCAM* | DS cell adhesion molecule | - | + | + | + | + | None | | Background-sensitive perinatal lethality associated with respiratory distress, altered C4 ventral root and pre-inspiratory neuron signaling, and abnormal response to hypercapnia. |
| *DVL3* | Dishevelled segment polarity protein 3 | - | + | - | + | + | Robinow syndrome, autosomal dominant 3 [MIM:601368] | | Postnatal lethality associated with respiratory distress, persistent truncus arteriosis, and double outlet right ventricle. |
| *DYRK1A* | Dual specificity tyrosine phosphorylation regulated kinase 1a | - | + | + | + | + | Mental retardation [MIM:600855] | | Embryonic growth delay and die during midgestation. Heterozygotes display reduced postnatal survival, postnatal growth retardation, microcephaly, behavioral and motor deficits, and altered neocortical pyramidal cell morphology. |
| EP300 | E1A binding protein p300 | + | + | - | + | + | Rubinstein-Taybi syndrome 2 [MIM:602700] | | Homozygotes for a targeted null mutation exhibit defects of the heart, lung, and small intestine and die at midgestation; heterozygotes also show some embryonic loss. Heterozygotes for an acetyltransferase-negative mutation die by the neonatal period. |
| EP400 | E1A binding protein p400 | + | - | - | - | - | None | | Die at E11.5 and display severe defects in yolk sac erythropoiesis, anemia, and a slight deformity of the neural tube. |
| *EPHB2* | EPH receptor B2 | - | + | - | + | + | None | | Abnormal axon guidance, circling, head bobbing, and hyperactivity. |
| *ERBIN* | Erbb2 interacting protein | - | + | - | - | + | None | | Impaired myelination, reduced nerve conduction, and hyporesponsiveness to tactile stimuli. |
| *FAM8A1* | Family with sequence similarity 8 member a1 | - | - | - | - | - | None | |  |
| *FOXP1* | Forkhead box p1 | - | + | + | - | - | Mental retardation with language impairment and with or without autistic features [MIM:605515] | | Embryonic lethality with abnormal outflow tract septation, ventricular septal defects, abnormal cardiac valve morphology, decreased and irregular heart rate, thin ventricular compact zone, and edema. |
| *FOXP3* | Forkhead box p3 | + | - | - | - | - | Immunodysregulation, polyendocrinopathy, and enteropathy, X-linked [MIM:300292] | | Hemizygous mutant males exhibit scaly skin, reddening and swelling of genital papilla, small undescended testes, depressed platelet and red cell counts, and lymphohistiocytic proliferation in various organs. Mutants die around weaning age. |
| FRYL | FRY like transcription coactivator | - | - | - | - | - | None | | Postnatal lethality and defects in kidney development; rare survivors display growth retardation, decreased body weight, and premature death associated with chronic hydronephrosis. |
| *GABRB3* | Gamma-aminobutyric acid type A receptor beta3 subunit | - | + | - | - | + | Epileptic encephalopathy, early infantile, 43 [MIM:137192] | | Die at birth with cleft palate. Survivors show delayed growth, reduced lifespan, seizures, ataxia, hyperactivity, hyper-responsiveness and reduced learning, mothering ability and rem sleep. |
| *GALNT18* | Polypeptide N-acetylgalactosaminyltransferase 18 | - | - | - | - | - | None | |  |
| *GIGYF2* | GRB10 interacting GYF protein 2 | - | - | - | - | - | {Parkinson disease 11} [MIM:612003] | | Mice homozygous for a knock-out allele exhibit neonatal and postnatal lethality. Mice heterozygous for a knock-out allele exhibit impaired motor coordination with motor neuron degeneration. |
| *GLI2* | GLI family zinc finger 2 | - | + | + | - | - | Holoprosencephaly 9; Culler-Jones syndrome, [MIM:165230] | | Skeletal malformations, absence of floorplate and foregut, lung and anorectal defects, and altered commissural neuron guidance. Most mutants die before embryonic day 18.5. |
| *GPRIN1* | G protein regulated inducer of neurite outgrowth 1 | - | + | - | + | - | None | | Decreased neutrophil cell number |
| *GPS1* | G protein pathway suppressor 1 | - | + | - | - | + | None | | Abnormal pericardium morphology  Abnormal embryo size  Abnormal liver size  Preweaning lethality, complete penetrance  Embryonic lethality prior to organogenesis |
| *GRB10* | Growth factor receptor bound protein 10 | - | + | + | - | - | None | | Maternal transmission of a mutant allele results in both fetal and placental overgrowth. Disproportionate overgrowth of the liver is observed. Paternal transmission of an allele lacking the differentially methylated region results in growth retardation. |
| *GRIA2* | Glutamate ionotropic receptor AMPA type subunit 2 | - | + | - | - | + | None | | Epilepsy, deficient dendritic architecture, altered exploratory behavior, impaired motor and learning performance, and increased mortality. |
| *GRIN1* | Glutamate ionotropic receptor NMDA type subunit 1 | - | + | - | - | + | Neurodevelopmental disorder with or without hyperkinetic movements and seizures, [MIM:138249] | | Null mutants lack whisker patterns in brain cortex, are ataxic and die neonatally of respiratory failure. Hypomorph mutants exhibit hyperactivity, stereotypy, and impaired social/sexual interactions. Mice homozygous for an ENU-induced allele exhibit abnormal behavior and neuron physiology. |
| *GRIN2A* | Glutamate ionotropic receptor NMDA type subunit 2A | - | + | - | - | + | Epilepsy, focal, with speech disorder and with or without mental retardation [MIM:138253] | | Jumpiness, mildly impaired long-term potentiation and spatial learning, increased locomotor activity and metabolism of dopamine and serotonin, and loss of analgesic tolerance after repeated morphine doses. |
| *GRIN2B* | Glutamate ionotropic receptor NMDA type subunit 2B | - | + | + | + | + | Epileptic encephalopathy, early infantile, 27; /Mental retardation [MIM:138252] | | Impairments in suckling, in hippocampal long term depression, and in pattern formation of trigeminal nucleus sensory afferent terminals. Mutants die shortly after birth. |
| *GTPBP4* | GTP binding protein 4 | - | - | - | - | - | None | |  |
| *HDAC9* | Histone deacetylase 9 | + | + | - | + | - | None | | Age dependent cardiac hypertrophy. |
| *HECTD1* | HECT domain E3 ubiquitin protein ligase 1 | - | - | - | - | - | None | | Exencephaly associated with impaired head mesenchyme development and neural tube closure, and show eye and cranial vault dysplasia. Homozygotes for another ENU-induced allele show congenital cardiovascular defects. |
| *HIVEP3* | Human immunodeficiency virus type I enhancer binding protein 3 | - | - | - | - | - | None | | Diminished IL-2 production by stimulated CD4 cells. Increased bone volume. |
| *HUWE1* | HECT, UBA and WWE domain containing 1, E3 ubiquitin protein ligase | + | + | - | + | + | Mental retardation, X-linked syndromic, Turner type [MIM:300697] | | Neonatal lethality, poorly developed dentate gyrus, small cerebellum, increased cortex density, and increased neuronal precursor cell proliferation. |
| *ILF2* | Interleukin enhancer binding factor 2 | - | - | - | - | - | None | | Embryonic lethality. |
| *IQGAP3* | IQ motif containing GTPase activating protein 3 | - | + | - | + | - | None | |  |
| *IQSEC2* | IQ motif and sec7 domain 2 | - | + | - | + | - | Mental retardation, X-linked 1/78 [MIM:300522] | |  |
| *ITPR1* | Inositol 1,4,5-trisphosphate receptor type 1 | - | + | - | + | + | Spinocerebellar ataxia; Gillespie syndrome [MIM:147265] | | Most die in utero, while survivors exhibit severe ataxia, seizures, and lethality by weaning age. |
| *KAT2B* | Lysine acetyltransferase 2B | + | + | + | - | - | None | | No abnormal phenotype. |
| KAT6A | Lysine acetyltransferase 6A | + | + | + | - | - | Mental retardation, autosomal dominant 32 [MIM:601408] | | Homozygous null mice display perinatal lethality, cyanosis, decreased hematopoietic progenitor cell numbers, and severely impaired spleen and thymus development, but are not anemic. Heterozygotes display strain background dependent reductions in fertility. |
| *KATNAL2* | Katanin catalytic subunit A1 like 2 | - | + | - | + | - | None | | Male sterility associated with defects in several aspects of spermatogenesis, including abnormalities in the initiation of sperm tail growth from the basal body, sperm head shaping, manchette movement and dissolution, acrosome attachment to the nucleus, and sperm release via spermiation. |
| *KCNH1* | Potassium voltage-gated channel subfamily H member 1 | - | + | - | - | + | Temple-Baraitser syndrome; Zimmermann-Laband syndrome 1 [MIM:603305] | | Decreased depressive-like response during tail suspension testing. Longer latency to move in haloperidol-treated mice and mild hyperactivity. |
| *KCNH3* | Potassium voltage-gated channel subfamily H member 3 | - | + | - | - | + | None | | Abnormal long term object recognition memory, spatial reference memory, spatial working memory, and long term potentiation.  Neuron hyperexcitability and seizures. |
| KDM5B | Lysine demethylase 5B | + | + | + | - | - | Mental retardation, autosomal recessive 65 [MIM:605393] | | Decreased body weight, background-sensitive premature mortality, decreased female fertility, delayed mammary gland development, decreased serum estradiol levels, and reduced mammary epithelial cell proliferation in early puberty. |
| KDM6B | Lysine demethylase 6B | + | + | - | - | + | None | | Perinatal death, thick alveolar septum, and absence of air space in the lungs. Another allele die neonatally displaying abnormal lung development, dwarfism, kyphosis, short limbs, and a severe delay in endochondral ossification. |
| KIAA0100 | KIAA0100 | - | - | - | - | - | None | |  |
| *KIRREL3* | Kirre like nephrin family adhesion molecule 3 | - | + | - | - | + | Mental retardation, autosomal dominant 4 [MIM:607761] | | Impaired accessory olfactory bulb formation with reduced coalescence of vomeronasal sensory neuron axons in the posterior accessory olfactory bulb, loss of male-male aggression and abnormal male sexual response to a male intruder mouse. |
| *KMT2A* | Lysine methyltransferase 2A | + | + | + | - | + | Wiedemann-Steiner syndrome [MIM:159555] | | Homozygotes for targeted null mutations die at embryonic day 11.5-14.5 with edematous bodies, petechiae, and hematopoietic insufficiency. Heterozygotes show reduced growth, hematopoietic abnormalities, and homeotic transformations of the axial skeleton. |
| *KMT2C* | Lysine methyltransferase 2C | + | + | + | - | - | Kleefstra syndrome 2 [MIM:606833] | | Partial embryonic lethality, delayed eyelid opening, postnatal growth retardation, impaired fertility in both sexes, and decreased proliferation of cultured mouse embryonic fibroblasts. |
| *KMT2E* | Lysine methyltransferase 2E | + | + | + | - | - | None | | Neonatal and postnatal lethality, reduced fertility and growth, and abnormal lymphopoiesis. |
| *KMT5B* | Lysine methyltransferase 5B | + | - | - | - | - | Mental retardation, autosomal dominant 51 [MIM:610881] | | Born at sub-Mendelian ratios, are smaller than control littermates, and die within a few hours of birth, probably due to alveolar defects. |
| *LAMC3* | Laminin subunit gamma 3 | - | + | - | - | - | Cortical malformations, occipital [MIM:604349] | | Abnormal amacrine cell morphology. |
| *LHX1* | Lim homeobox 1 | - | + | + | - | - | None | | Small, fail to develop head structures anterior to rhombomere 3 in the hindbrain, lack kidneys and gonads, and show aberrant trajectories of limb motor axons. Most mutants die around embryonic day 10. |
| LRRFIP1 | LRR binding FLII interacting protein 1 | - | - | - | - | - | None | |  |
| *LZTR1* | Leucine zipper like transcription regulator 1 | - | - | - | - | - | Noonan syndrome 10; {Schwannomatosis-2, susceptibility to} [MIM:600574] | | Mice homozygous for a null allele die between E17.5 and birth. Heterozygotes exhibit Noonan syndrome phenotypes, including decreased weight and facial dysmorphia in males and eccentric hypertrophy, enlarged myocardial fibers and premature death in both sexes. |
| *MAGEL2* | Mage family member l2 | - | + | + | + | - | Schaaf-Yang syndrome [MIM:605283] | | Mice heterozygous for a null allele that is inherited paternally exhibit some postnatal lethality, reduced male fertility, abnormal circadian rhythm, and hypoactivity. Mice heterozygous for another paternal knock-out allele exhibit 50% neonatal lethality associated with weak suckling activity. |
| *MAP3K1* | Mitogen-activated protein kinase kinase kinase 1 | - | + | - | - | - | 46XY sex reversal 6 [MIM:600982] | | Mice homozygous for a spontaneous allele are born with one or both eyes open, defects in eye morphology, and defects in ear morphology and function. Mice homozygous for a knock-out allele are born with open eyes and exhibit increased response to aortic banding and blood vessel healing. |
| *MECP2* | Methyl-CpG Binding Protein 2 | + | + | + | + | - | Rett syndrome [MIM:300005] | | Female mice homozygous or male mice hemizygous for a null allele exhibit premature death, behavioral and neurological abnormalities, abnormal nervous system phenotypes, abnormal breathing, and abnormal hearing. Heterozygous mice exhibit similar behavioral and neurological abnormalities. |
| *MED13L* | Mediator complex subunit 13 like | - | + | + | - | - | Mental retardation and distinctive facial features with or without cardiac defects [MIM:608771] | |  |
| *MFRP* | Membrane frizzled-related protein | - | - | - | - | - | Microphthalmia, isolated 5; Nanophthalmos 2 [MIM:606227] | | Mutations produce mice having small, white retinal spots and progressive photoreceptor degeneration. |
| *MKI67* | Marker of proliferation Ki-67 | - | - | - | - | - | None | | no abnormal phenotype detected |
| *MOV10* | Mov10 RISC complex RNA helicase | - | + | - | + | + | None | | Homozygous knockout is embryonic lethal. Heterozygous knockout leads to reduced dendritic branching of neurons, which affects anxiety- and/or activity-related behavior. |
| *MYH9* | Myosin heavy chain 9 | - | + | - | + | - | None | | Homozygous null mice display embryonic lethality. Heterozygous null mice display hearing loss with incomplete penetrance. Mice homozygous or heterozygous for one of several knock-in alleles exhibit macrothrombocytopenia, nephritis, cataracts and deafness. |
| *MYT1L* | Myelin transcription factor 1 like | - | + | + | - | - | Mental retardation, autosomal dominant 39 [MIM:613084] | | decreased circulating glycerol level  increased fluid intake |
| *NAA15* | N(Alpha)-Acetyltransferase 15, NatA Auxiliary Subunit | - | + | + | - | - | Mental retardation, autosomal dominant 50 [MIM:608000] | |  |
| *NAT8L* | N-acetyltransferase 8 like | - | + | - | + | - | N-acetylaspartate deficiency: truncal ataxia, marked developmental delay, seizures, and secondary microcephaly [MIM:610647] | | Abnormal responses to novelty and decreased social investigation in a novel environment. |
| *NAV2* | Neuron navigator 2 | - | + | - | + | - | None | | Impaired olfaction and hearing, increased latency in a hot plate test, degeneration of the optic nerve, decreased exploration in new environments, and weight loss. |
| *NBEA* | Neurobeachin | - | + | - | - | + | None | | Die shortly after birth, are cyanotic, and exhibit no response to tactile stimuli, no spontaneous movement, and impaired CNS synaptic transmission. |
| *NCKAP1* | NCK associated protein 1 | - | + | - | + | - | None | | Growth arrest at midgestation, an open neural tube, cardia bifida, defective foregut development, defects in endoderm and mesoderm migration and sometimes duplication of the anteroposterior body axis. |
| *NCOR1* | Nuclear receptor corepressor 1 | + | + | + | - | - | None | | Mice homozygous for a targeted mutation in this gene exhibit embryonic lethality with erythrocytic, thymocytic and central nervous system development abnormalities. Mice homozygous for a hypomorphic allele exhibit increased thyroid hormone sensitivity under hypothyroid conditions. |
| *NEMF* | Nuclear export mediator factor | - | - | - | - | - | None | |  |
| *NFIA* | Nuclear factor I A | - | + | - | - | - | Brain malformations and urinary tract defects [MIM:600727] | | Homozygous null mice display perinatal lethality, hydrocephalus, agenesis of the corpus callosum and hippocampal commissure. Fertility is surviving homozygotes is compromised. A decrease in the number of heterozygous animals is associated with a maternal effect. |
| NIN | Ninein | - | + | - | + | - | ?Seckel syndrome 7 [MIM:614851] | | Partial pre- and postnatal lethality, thinner skin, delayed skin barrier formation, impaired skin barrier function, impaired differentiation of suprabasal cells, and defects in mitotic spindle orientation, desmosome assembly and lamellar body secretion. |
| *NISCH* | Nischarin | - | + | + | + | - | None | | Hearing loss associated with increased susceptibility to otitis media. |
| *NLGN3* | Neuroligin 3 | - | + | - | - | + | {Autism susceptibility, X-linked 1} [MIM:300336] | | Homozygous null mice show impaired context and cued conditioning, hyperactivity, altered social behavior, less vocalization, smaller brains, and impaired olfaction. Males carrying a knock-in allele show impaired social interaction, and enhanced spatial learning and inhibitory synaptic transmission. |
| *NLGN4X* | Neuroligin 4 X-linked | - | + | - | - | + | Mental retardation, X-linked [MIM:300427] | |  |
| NOTCH1 | Notch 1 | - | + | + | + | + | Aortic valve disease 1; Adams-Oliver syndrome 5 [MIM:190198] | | Defects in embryonic development resulting in lethality at some point in organogenesis. Lethal phenotype may be affected by genetic background. |
| *NR2E3* | Nuclear receptor subfamily 2 group E member 3 | - | - | - | - | - | Enhanced S-cone syndrome [MIM:604485] | | Mice homozygous for a null mutation exhibit rossettes and a reduced number of nuclei in the retinal outer nuclear layer. |
| *NRXN1* | Neurexin 1 | - | + | - | - | + | Pitt-Hopkins-like syndrome 2 [MIM:600565] | | Reduced Ca(2+)-dependent binding of alpha-latrotoxin to brain membranes. Isolated synaptosomes display only a small reduction in alpha-latrotoxin -triggered glutamate release in the absence of Ca(2+) but show a major decrease in the presence of Ca(2+). |
| NSD2 | Nuclear receptor binding set domain protein 2 | + | + | + | - | - | Wolf-hirschhorn syndrome candidate 1[MIM:602952] | | Reduced fetal size, failed sternum ossification, cleft palate, atrial and ventricular septal defects, stunted growth and postnatal death. Some heterozygotes show severe growth defects, malocclusion, delayed sternum ossification and hypoplasia of the septum secundum. |
| *NTM* | Neurotrimin | - | + | - | + | - | None | | Impaired behavioral response to amphetamine and a deficit in emotional learning in the active avoidance ask. |
| *NTNG1* | Netrin G1 | - | + | - | - | + | None | | Survive into adulthood with no major alterations in gross brain cytoarchitecture or axonal projection. |
| *NUAK1* | NUAK family kinase 1 | - | + | - | + | - | None | | Die perinatally displaying omphalocele with a failure in closure of the secondary body wall. No gross morphological defects are detected in brain. |
| *PARD3B* | Par-3 family cell polarity regulator beta | - | - | - | - | - | None | | Abnormal behavior  Abnormal heart left ventricle morphology  Abnormal lens morphology  Hyperactivity |
| *PASK* | PAS domain containing serine/threonine kinase | - | + | + | - | - | None | | Resistance to diet-induced obesity, impaired glucose stimulated insulin secretion, abnormal energy balance, and abnormalities in hypoxia induced changes in ventialtion. |
| *PAX5* | Paired box 5 | + | + | + | - | - | {Leukemia, acute lymphoblastic, susceptibility to, 3} [MIM:167414] | | Impaired development of the midbrain resulting in a reduced inferior colliculus and an altered cerebellar folial pattern, failure of B cell differentiation, runting, and high postnatal mortality with few survivors. |
| *PCOLCE* | Procollagen c-endopeptidase enhancer | - | - | - | - | - | None | | Thickened cortical and trabecular bone and abnormal collagen fibrils in both mineralized and nonmineralized tissues. |
| *PHF2* | PHD finger protein 2 | + | + | + | - | - | None | | Partial postnatal lethality, decreased body weight, decreased adipocity and impaired adipogenesis. |
| *PHF3* | PHD finger protein 3 | - | - | - | - | - | None | |  |
| *PHF7* | PHD finger protein 7 | - | - | - | - | - | None | | Male infertility |
| *PHIP* | Pleckstrin homology domain interacting protein | - | - | - | - | - | Developmental delay, intellectual disability, obesity, and dysmorphic features [MIM:612870] | | Postnatal and premature lethality associated with reduced body size, small myocardial cells and hepatocytes, hypoglycemia, increased insulin sensitivity, and reduced cell growth. |
| *PIK3CA* | Phosphatidylinositol-4,5-bisphosphate 3-kinase catalytic subunit alpha | - | + | + | - | - | Megalencephaly-capillary malformation-polymicrogyria syndrome; Cowden syndrome 5 [MIM:171834] | | Embryonic death associated with growth retardation, vascular defects and hemorrhage. Surviving mice homozygous for a knock-in allele show impaired lymphangiogenesis, ascites, reduced weight, and resistance to ras-driven skin tumorigenesis. |
| *PLXNA3* | Plexin A3 | - | + | - | + | - | None | | Misdirected axons in the hippocampus. They are, otherwise, indistinguishable from wild-type mice. |
| *PLXNB1* | Plexin B1 | - | + | - | + | + | None | | Viable and fertile and show no apparent defects in development, adult histology or basic functional parameters. However, a transitory renal phenotype, characterized by increased ureteric branching and enlarged kidneys, is noted over early stages of renal development. |
| *POGZ* | Pogo transposable element derived with ZNF domain | - | + | - | + | + | White-Sutton syndrome [MIM:614787] | | Lethality during organogenesis, fetal development and preweaning associated with fetal liver hypoplasia, small fetus size and anemia. |
| *PPM1D* | Protein phosphatase, Mg2+/Mn2+ dependent 1D | - | + | - | + | - | Intellectual developmental disorder with gastrointestinal difficulties and high pain threshold [MIM:605100] | | Some embryonic lethality. Surviving males have variable abnormalities including runting, reproductive organ atrophy with associated reduced fertility, and reduced life span. Both genders have increased susceptibility to viral infection and reduced lymphocyte function. |
| *PPP2R5D* | Protein phosphatase 2 regulatory subunit B'delta | - | - | - | - | - | Mental retardation, autosomal dominant 35 [MIM:601646] | | Mice homozygous for a gene-trap allele exhibit lethality, while heterozygous mice display decreased prepulse inhibition. Mice homozygous for a targeted knock-out allele exhibit decreased thermal nociception threshold, impaired coordination, and increasedlatency to removing an adhesive sticker. |
| *PROX2* | Prospero homeobox 2 | - | - | - | - | - | None | | Normal growth, survival and fertility, with no detectable defects in eye structure or horizontal cell development in the retina. |
| *PSEN2* | Presenilin 2 | - | + | - | - | + | Alzheimer disease-4 ; Cardiomyopathy, dilated, 1V [MIM:600759] | | Viable and fertile, but older mutants develop mild pulmonary fibrosis and hemorrhage. |
| PSMD12 | Proteasome 26S subunit, non-ATPase 12 | - | - | - | - | - | Stankiewicz-Isidor syndrome [MIM:604450] | |  |
| *PTEN* | Phosphatase and tensin homolog | - | + | + | + | + | Macrocephaly/autism syndrome [MIM:601728] | | Homozygous null mutants die by E9.5 with abnormally patterned enlarged brains and defective placentas. Heterozygotes develop a range of neoplasms. Conditional mutants demonstrate effects on basic processes of proliferation, differentiation and apoptosis. |
| *PTK7* | Protein tyrosine kinase 7 (inactive) | - | + | - | + | - | None | | Mice homozygous for a gene trapped allele die perinatally with defects in neural tube closure and planar cell polarity in the ear. ENU-induced mutant mice show omphalocele, impaired neural tube, heart and lung development, rib defects, polydactyly, failed eyelid closure and altered cell polarity. |
| *PTPN11* | Protein tyrosine phosphatase, non-receptor type 11 | - | + | + | + | + | Noonan syndrome 1; LEOPARD syndrome 1 [MIM:176876] | | Homozygous null mutants exhibit abnormal mesoderm patterning leading to a failure of gastrulation and death by embryonic day 10.5. In heterozygous state the null mutant acts as a dominant enhancer of a mild epidermal growth factor receptor mutation. Conditional KO in the eye results in severe retinal degeneration. |
| *RAB2A* | Rab2a, member RAS oncogene family | - | + | - | - | - | None | | Edema  Decreased vertical activity  Preweaning lethality, complete penetrance  Increased freezing behavior |
| *RALGAPB* | Ral GTPase activating protein non-catalytic beta subunit | - | - | - | - | - | None | | Decreased circulating insulin level,  Preweaning lethality, complete penetrance |
| *RANBP2* | RAN binding protein 2 | - | + | + | + | - | {Encephalopathy, acute, infection-induced, 3, susceptibility to} [MIM:601181] | | Mice homozygous for a gene trap allele display embryonic lethality. Heterozygous mice on some backgrounds display reduced ATP levels in the CNS, decreased glucose clearance, decreased weight gain on a high fat diet, and reduced scotopic responses. |
| *RELN* | Reelin | - | + | + | + | + | Lissencephaly 2 (Norman-Roberts type), {Epilepsy, familial temporal lobe, 7} [MIM:600514] | | Homozygotes for most spontaneous or ENU-induced mutations show impaired righting responses, ataxia, tremors, and cerebellum and hippocampus abnormalities. Some mutants show postnatal or premature death and decreased body size while others have abnormal retinas or olfactory bulbs or infertility. |
| *RIMBP2* | RIMS binding protein 2 | - | - | - | - | - | None | | Mild neurological phenotype with changes in the synaptic transmission and plasticity of hippocampal neurons. |
| *RIMS1* | Regulating synaptic membrane exocytosis 1 | - | + | - | - | + | Cone-rod dystrophy 7 [MIM:606629] | | Defects in maternal care and abnormalities in synaptic transmission in the central nervous system. |
| *RNF38* | Ring finger protein 38 | - | - | - | - | - | None | |  |
| *RPL19* | Ribosomal protein l19 | - | - | - | - | - | None | |  |
| *SCN1A* | Sodium voltage-gated channel alpha subunit 1 | - | + | - | - | + | Epilepsy [MIM:182389] | | Homozygous null mice show postnatal lethality, seizures and behavioral deficits whereas heterozygotes die prematurely with seizures and abnormal electrophysiology. In addition, knock-in mice exhibit increased susceptibility to febrile and flurothyl-induced seizures, and reduced inhibitory signaling. |
| *SCN2A* | Sodium voltage-gated channel alpha subunit 2 | - | + | - | + | - | Seizures, Epileptic encephalopathy, early infantile, 11 [MIM:182390] | | Excess neuronal apoptosis (especially in the brainstem), reduced neuronal sodium channel currents in vitro, and severe hypoxia resulting in neonatal lethality. |
| *SETBP1* | SET binding protein 1 | + | + | + | - | - | Schinzel-Giedion midface retraction syndrome [MIM:611060] | |  |
| *SETD2* | SET domain containing 2 | + | + | + | + | - | Luscan-Lumish syndrome [MIM:612778] | | Impaired embryonic vascular remodeling in the embryo proper, yolk sac, and placenta that leads to death around E10.5. |
| *SETD5* | SET domain containing 5 | + | - | - | - | - | Mental retardation [MIM:615743] | | Embryonic or fetal lethality. Observed phenotypes include embryonic growth retardation, impaired neural tube formation, somitogenesis and cardiac development, abnormal vasculogenesis in embryos, yolk sacs and placentas, hemorrhage and increased apoptosis. |
| *SHANK1* | SH3 and multiple ankyrin repeat domains 1 | - | + | - | + | + | None | | Smaller pyramidal neuron dendritic spines, smaller and thinner postsynaptic density of central excitatory synapses, weaker synaptic transmission, increased anxiety-related behavior, and impaired contextual fearmemory, but enhanced spatial learning. |
| *SHANK2* | SH3 and multiple ankyrin repeat domains 2 | - | + | - | + | + | {Autism susceptibility 17} [MIM:603290] | | Hyperactivity and abnormal social behavior. Partial postnal lethality and limb grasping. |
| *SIN3A* | SIN3 transcription regulator family member a | + | + | + | + | - | Witteveen-kolk syndrome [MIM:607776 ] | | Targeted disruption of this gene results in early embryonic lethality. Homozygous null MEFs display poor cell proliferation, reduced S-phase and increased G2/M fractions, a block in DNA replication, and enhanced apoptosis; however, no increase in chromosomal instability is observed. |
| *SLC6A1* | Solute carrier family 6 member 1 | - | + | - | - | + | Myoclonic-atonic epilepsy [MIM:137165 ] | | Homozygous hypomorphic mice display abnormal inhibitory postsynaptic currents, and abnormal GABA uptake and release. Null mice show hyperactivity and various behavioral abnormalities, as well as an aversion to bitter taste. |
| *SLC6A10P* | Solute carrier family 6 member 10, pseudogene | - | - | - | - | - | None | |  |
| *SLC6A8* | Solute carrier family 6 member 8 | - | + | - | - | + | Cerebral creatine deficiency syndrome 1 [MIM:300036] | | Male mice hemizygous for a targeted allele exhibit decreased body weight, decreased creatine concentrations, impaired short term object recognition, impaired contextual conditioning, altered locomotor activity, and increased serotonine levels in the brain. |
| *SLC9A3* | Solute carrier family 9 member A3 | - | - | - | - | - | Diarrhea 8, secretory sodium, congenital [MIM:182307] | | Diarrhea associated with defects of renal and intestinal absorption. Males are infertile. |
| *SLITRK5* | SLIT and NTRK like family member 5 | - | + | - | - | + | None | | Abnormal medium spiny neuron morphology and exhibit behavioral abnormalities. |
| *SMAD2* | SMAD family member 2 | - | + | + | + | - | None | | Homozygous mutant embryos die at day 6.5-8.5 with multiple defects, including failed gastrulation, lack of mesoderm, visceral endoderm dysfunction and failure to form anterior-posterior axis. Heterozygotes may show gastrulation defects and lack mandible or eyes. |
| *SMAD4* | SMAD family member 4 | - | + | + | - | - | Myhre syndrome: pre- and postnatal short stature, brachydactyly, facial dysmorphism, thick skin, muscle hypertrophy, deafness, and developmental delay [MIM:600993] | | Homozygotes for targeted null mutations exhibit impaired formation of extraembryonic membrane and endoderm and die prior to gastrulation. Heterozygotes develop polyposis of the glandular stomach and duodenum. |
| *SMARCC2* | SWI/SNF related, matrix associated, actin dependent regulator of chromatin subfamily c member 2 | + | + | + | - | - | None | | Mice homozygous for a targeted allele exhibit a slight increase in embryo weight at E13.5 and die shortly after birth (P0-P3). Mice homozygous for a conditional allele activated in the brain exhibit reduced cerebral cortical size and thickness. |
| *SMC3* | Structural maintenance of chromosomes 3 | - | + | - | + | - | Cornelia de Lange syndrome 3 [MIM:606062] | | Mice homozygous for a knock-out allele exhibit complete embryonic lethality. Mice heterozygous for this allele exhibit partial postnatal lethality, decreased body weight, abnormal craniofacial morphology, and increased T cell number. |
| *SPAST* | Spastin | - | + | - | + | - | Spastic paraplegia 4 [MIM:604277] | | Mice homozygous for a mutation in this gene are sterile and display progressive axonopathy with focal axonal swellings and late onset gait abnormalities. |
| *SPP2* | Secreted phosphoprotein 2 | - | - | - | - | - | None | |  |
| SPRED2 | Sprouty related EVH1 domain containing 2 | - | + | + | + | + | None | | Homozygous null mice are fertile and display increased hematopoietic cell formation in culture. |
| SPTBN1 | Spectrin beta, non-erythrocytic 1 | - | + | - | + | - | None | | Homozygous inactivation of this gene leads to mid-gestational lethality due to gastrointestinal, liver, neural, and cardiac defects, whereas heterozygotes survive until adulthood and spontaneously develop cancers in several organs. |
| *SRCAP* | Snf2 related CREBBP activator protein | + | - | - | - | - | Floating-Harbor syndrome [MIM:611421] | |  |
| *SRGAP2* | SLIT-ROBO Rho GTPase activating protein 2 | - | + | + | - | + | None | | Mice homozygous for a hypomorphic gene trap allele are born at below the expected Mendelian ratio, but are otherwise viable. Layer 5 cortical pyramidal neurons exhibit an increased density of dendritic spines with a decreased spine head width and increased length of spine necks. |
| *SRGAP3* | SLIT-ROBO Rho gtpase Activating Protein 3 | - | + | + | - | - | None | | Mice homozygous for a knock-out allele exhibit a reduction in spine density in the brain CA1 and cortical layers IV/V. Mice homozygous for a different knock-out allele exhibit a neurodevelopment disorder with schizophrenia-related intermediate phenotypes. |
| *SRM* | Spermidine synthase | - | - | - | - | - | None | |  |
| SRRM2 | Serine/arginine repetitive matrix 2 | - | - | - | - | - | None | |  |
| *ST3GAL6* | ST3 beta-galactoside alpha-2,3-sialyltransferase 6 | - | - | - | - | - | None | | Mice homozygous for a knock-out allele exhibit modest impairment in leukocyte rolling and neutrophil recruitment. |
| *STAG1* | Stromal antigen 1 | - | - | - | - | - | Mental retardation, autosomal dominant 47 [MIM:604358 ] | | Mouse embryos homozygous for a null mutation show developmental delay and die before birth. Heterozygous animals have shorter lifespan and earlier onset of tumourigenesis. |
| *STXBP1* | Syntaxin binding protein 1 | - | + | - | + | + | Epilepsy encephalopathy, early infantile, 4 [MIM:602926] | | Mice homozygous for a null allele exhibit total loss of neurotransmitter secretion from synaptic vesicles throughout development and massive neuron apoptosis after initial synaptogenesis, leading to widespread neurodegeneration and complete neonatal lethality. |
| *STXBP5* | Syntaxin binding protein 5 | - | + | - | + | + | None | | Mice homozygous for a null allele exhibit some background sensitive prenatal lethality and increased synaptic transmission. |
| *SUPT16H* | SPT16 homolog, facilitates chromatin remodeling subunit | + | - | - | - | - | None | |  |
| *SVIL* | Supervillin | - | - | - | - | - | None | | Mice homozygous for a knock-out allele exhibit enhanched adhesion and thrombus formation. |
| *SYNCRIP* | Synaptotagmin binding cytoplasmic rna interacting protein | - | + | - | - | + | None | |  |
| *SYNGAP1* | Synaptic Ras GTPase activating protein 1 | - | + | - | + | + | Mental retardation, autosomal dominant 5 [MIM:603384] | | Postnatal lethality, and by P3-P4, exhibit small body size and brain, reduced movement and do not feed. |
| *TANC2* | Tetratricopeptide repeat, ankyrin repeat and coiled-coil containing 2 | - | + | - | + | + | None | | Die prior to E12. |
| *TBL1XR1* | Transducin beta like 1 X-linked receptor 1 | + | - | - | - | - | Pierpont syndrome: Plantar lipomatosis, unusual facies, developmental delay; Mental retardation, autosomal dominant 42 [MIM:608628] | | Mice homozygous for a conditional allele activated in adipose tissue exhibit increased body weight, and total body fat and increased susceptibility to diet-induced obesity and impaired glucose homeostasis. |
| *TBR1* | T-box, brain 1 | - | + | + | + | - | ID phenotype (PMID: 24458984) | | Mice homozygous for a targeted null allele fail to feed and die on the second postnatal day displaying disrupted forebrain morphology and a hypoplastic olfactory bulb that lacks normal mitral and tufted cells and shows a striking reduction in mature olfactory bulb projection neurons. |
| *TCF4* | Transcription factor 4 | - | + | + | + | + | Pitt-Hopkins syndrome [MIM:602272] | | Animals homozygous for a targeted mutation exhibit intestinal epithelia abnormalities and die shortly after birth. Mice heterozygous for some mutations display abnormalities in glucose homeostasis. |
| *TCF7L2* | Transcription factor 7 like 2 | - | + | + | + | - | None | | Animals homozygous for a targeted mutation exhibit intestinal epithelia abnormalities and die shortly after birth. Mice heterozygous for some mutations display abnormalities in glucose homeostasis. |
| TECTA | Tectorin alpha | - | - | - | - | - | Deafness [MIM:602574] | | Homozygous null mice exhibit a tectorial membrane that is detached from the cochlear epithelium. Though the basilar membranes of mutant mice are tuned, sensitivity is attenuated. Mice with an Y1870C mutation have a disrupted tectorial membrane, elevated neural thresholds and broadened neural tuning. |
| TLK2 | Tousled like kinase 2 | + | - | - | - | - | Mental retardation, autosomal dominant 57 [MIM:608439] | | Late embryonic lethality due to placental failure. Observed phenotypes include a small, hypocellular and poorly vascularized placenta with a disorganized labyrinth, and reduced numbers of syncytiotrophoblast, spongiotrophoblast and trophoblastic giant cells. |
| *TNRC18* | Trinucleotide repeat containing 18 | - | - | - | - | - | None | |  |
| *TNRC6B* | Trinucleotide repeat containing 6B | - | - | - | - | - | None | | Neonatal and postnatal lethality with decreased body weight and infertility. |
| *TRIO* | Trio Rho guanine nucleotide exchange factor | - | + | - | + | + | Mental retardation, autosomal dominant 44 [MIM:601893] | | Homozygous mutant mice die during late embryonic development or shortly after birth. They exhibit abnormal skeletal myogenesis and display aberrant organization within the hippocampus and olfactory bulb. |
| *TRIP12* | Thyroid hormone receptor interactor 12 | + | - | - | - | - | Mental retardation, autosomal dominant 49 [MIM:604506] | | Complete embryonic lethality during organogenesis associated with embryonic growth retardation and abnormal placenta development. |
| *TRRAP* | Transformation/transcription domain associated protein | + | + | + | - | - | None | | Homozygous embryos die prior to E3.5 and exhibit embryonic and extraembryonic tissue disorganization. Mitotic abnormalities were also noted in homozygous cells. |
| *TSC2* | TSC complex subunit 2 | - | + | + | + | + | Tuberous sclerosis-2 [MIM:191092] | | Homozygous null mutants exhibit liver hypoplasia, open neural tube, thickened myocardium and die by embryonic day 9.5-12.5. Heterozygotes develop renal cystadenomas, liver hemangiomas (sometimes resulting in fatal bleeding) and lung adenomas. |
| *TUBA1A* | Tubulin alpha 1a | - | + | + | - | - | Lissencephaly 3 [MIM:602529] | | Heterozygous mutation of this gene results in hyperactivity, reduced anxiety, impaired spatial working memory, and abnormalities in the laminar architecture of the hippocampus and cortex, accompanied by impaired neuronal migration. |
| *UBN2* | Ubinuclein 2 | - | - | - | - | - | None | |  |
| UBR3 | Ubiquitin protein ligase E3 component n-recognin 3 | - | - | - | - | - | None | | Homozygous null mice obtained on a coisogenic 129S1 background die early in embryogenesis while those on a mixed 129S1/B6 background are born at a slightly reduced frequency. On a congenic C57BL/6 background, homozygotes display neonatal lethality, impaired suckling and female behavioral anosmia. |
| *UIMC1* | Ubiquitin interaction motif containing 1 | + | - | - | - | - | None | | Premature death due to B-cell lymphomas and abnormal DNA repair. |
| *UNC80* | Unc-80 homolog, NALCN channel complex subunit | - | + | - | - | + | Hypotonia, infantile, with psychomotor retardation and characteristic facies 2 [MIM:612636] | |  |
| *VCP* | Valosin containing protein | - | + | + | + | - | Neurodegenerative phenotypes [MIM:601023] | | Homozygous mutation of this gene results in lethality before weaning. Mice homozygous for a knock-in allele exhibit progressive muscle weakness, myopathy, decreased bone density, increased osteoclast genesis, and seizures. |
| *WAC* | WW domain containing adaptor with coiled-coil | + | - | - | - | - | Desanto-Shinawi syndrome [MIM:615049] | |  |
| *WDFY3* | WD repeat and FYVE domain containing 3 | - | + | + | + | - | ?Microcephaly 18, primary, autosomal dominant [MIM:617485] | | Mice homozygous for hypomorphic mutations of this gene exhibit perinatal lethality, altered neural progenitor divisions and neuronal migration, a regionally enlarged cerebral cortex, and focal cortical dysplasias. |
| WDR33 | WD repeat domain 33 | - | - | - | - | - | None | |  |
| *WDR45* | WD repeat domain 45 | - | + | - | + | - | Neurodegeneration with brain iron accumulation 5 [MIM:300526] | | Mice homozygous for a conditional allele activated in neurons exhibit impaired autophagy, axonal swelling and degeneration and impaired learning and memory function. |
| *WNT7B* | Wnt family member 7b | - | + | + | + | - | None | | Homozygous null embryos die at midgestational stages due to placental abnormalities involving the fusion of the chorion and allantois. Mice homozygous for a truncated allele display neonatal lethality, respiratory failure, and lung hemorrhage. |
| *WNT9A* | Wnt family member 9A | - | + | + | - | - | None | | Homozygous inactivation of this gene results in neonatal lethality, altered chondrocyte maturation, cranial defects, and skeletal abnormalities including shortened appendicular long bones, partial joint fusions of carpal and tarsal elements, and chondroid metaplasia in synovial and fibrous joints. |
| *YTHDC1* | YTH domain containing 1 | - | - | - | - | - | None | | Mice homozygous for a null allele die between E8.5 and E11.5. Mice homozygous for a cconditional allele activated after embryonic development exhibit infertility with defects in spermatogenesis and oogenesis associated with RNA granules and altered transcription in oocytes. |
| *ZBTB45* | Zinc finger and BTB domain containing 45 | - | + | + | - | - | None | |  |
| ZC3H14 | Zinc finger CCCH-type containing 14 | - | + | - | + | + | Mental retardation, autosomal recessive 56 [MIM:613279] | | Impaired spatial working memory, enlarged anterior lateral ventricles in the brain, small testes and reduced litter size. |
| *ZC3H4* | Zinc finger CCCH-type containing 4 | - | - | - | - | - | None | | Complete embryonic lethality between implantation and somite formation and failure of blastocysts to hatch from the zona pellucida and form typical outgrowth colonies. |
| *ZFHX3* | Zinc finger homeobox 3 | - | + | + | - | - | None | | Mice homozygous for a gene trapped allele exhibit normal initial pituitary development but reduced GH and TSH-beta staining within the pituitary by E17.5. Mice homozygous for a knock-out allele exhibit prenatal lethality. Mice heterozygous for the same allele exhibit partial postnatal lethality, decreased body size and prolonged conception time. |
| *ZMYM2* | Zinc finger MYM-type containing 2 | - | + | + | - | - | None | | Exhibit prenatal lethality. |
| *ZMYND11* | Zinc finger MYND-type containing 11 | + | - | - | - | - | Mental retardation, autosomal dominant 30 [MIN:608668] | | Multiple abnormalities in including nervous and cardiovascular system. |
| *ZNF292* | Zinc finger protein 292 | - | - | - | - | - | None | |  |

**Supplementary Table S3**. Damaging *de novo* variants in the 3 gene ontologies with and without DYRK1A, CHD7 and PTPN11 genes

|  |  | **DDD Plus Study-Sifrim 2016**  **(N=1039)** | | | | **PCGC-Jin 2017**  **(N=2645)** | | | | **Combined CHD**  **(N=3684)** | | | |
| --- | --- | --- | --- | --- | --- | --- | --- | --- | --- | --- | --- | --- | --- |
|  | **Gene Set** | Allele Count | OR | 95% CI | p-value | Allele Count | OR | 95% CI | p-value | Allele Count | OR | 95% CI | p-value |
|  | **NDD** | 54 | 4.75 | 2.80, 8.06 | 4.64E-10 | 72 | 2.35 | 1.41, 3.91 | 6.40E-04 | 126 | 3.00 | 1.85, 4.88 | 9.48E-07 |
| **Excluded** | **Connectome** | 45 | 6.24 | 3.29, 11.8 | 3.42E-10 | 53 | 2.73 | 1.46, 5.13 | 7.62E-04 | 98 | 3.69 | 2.02, 6.73 | 1.06E-06 |
|  | **Chromatin Modifiers** | 24 | 5.62 | 2.41, 13.1 | 1.56E-05 | 19 | 1.67 | 0.70, 3.97 | 0.31 | 43 | 2.74 | 1.23, 6.11 | 0.0085 |
|  | **NDD** | 67 | 5.97 | 2.79, 7.67 | 4.81E-14 | 95 | 3.13 | 1.90, 5.14 | 7.10E-07 | 162 | 3.89 | 2.41, 6.28 | 7.80E-11 |
| **Included** | **Connectome** | 58 | 8.14 | 4.35, 15.2 | 1.38E-14 | 76 | 3.95 | 2.14, 7.28 | 5.99E-07 | 134 | 5.08 | 2.81, 9.20 | 6.30E-11 |
|  | **Chromatin Modifiers** | 27 | 6.33 | 2.75, 14.6 | 1.13E-06 | 33 | 2.91 | 1.28, 6.58 | 0.0080 | 60 | 3.84 | 1.75, 8.42 | 1.44E-04 |

**Supplementary Table S4.** No significant gene enrichment with *de novo* damaging variants from the two sets of random selected genes (n=229) within the CHD cases (p-value cutoff <2.18E-04, statistical significance after Bonferroni correction). The gene symbols were random selected from ~20,000 unique genes of human genome by Microsoft Excel RAND function.

| **Random Group1** | **Mutation Rate** | **Size** | **Observed** | **Expected** | **P-value** |
| --- | --- | --- | --- | --- | --- |
| LZTR1 | 4.81E-05 | 2544 | 2 | 0.90 | 4.50E-03 |
| LEF1 | 1.89E-05 | 1302 | 1 | 0.46 | 3.78E-02 |
| BBS2 | 2.97E-05 | 2183 | 1 | 0.77 | 5.87E-02 |
| CRYAA | 3.05E-05 | 2554 | 1 | 0.91 | 6.02E-02 |
| UHRF2 | 3.13E-05 | 2425 | 1 | 0.86 | 6.18E-02 |
| FOXM1 | 3.40E-05 | 2406 | 1 | 0.85 | 6.69E-02 |
| FGFR2 | 3.74E-05 | 2730 | 1 | 0.97 | 7.34E-02 |
| PPIP5K1 | 5.86E-05 | 4292 | 1 | 1.52 | 1.13E-01 |
| ARHGAP35 | 6.42E-05 | 4506 | 1 | 1.60 | 1.23E-01 |
| SCN5A | 0.000103 | 6083 | 1 | 2.16 | 1.89E-01 |

| **Random Group2** | **Mutation Rate** | **Size** | **Observed** | **Expected** | **P-value** |
| --- | --- | --- | --- | --- | --- |
| *POGZ* | 5.66E-05 | 4179 | 3 | 1.73 | 2.35E-04 |
| *CTNNB1* | 2.99E-05 | 2332 | 2 | 0.97 | 1.78E-03 |
| *DUSP23* | 3.04E-06 | 455 | 1 | 0.19 | 6.18E-03 |
| *HHLA3* | 6.00E-06 | 452 | 1 | 0.19 | 1.22E-02 |
| *TAGLN* | 9.21E-06 | 610 | 1 | 0.25 | 1.86E-02 |
| *DSCAML1* | 1.23E-04 | 6300 | 2 | 2.61 | 2.66E-02 |
| *ZNF174* | 1.59E-05 | 1307 | 1 | 0.54 | 3.20E-02 |
| *GC* | 1.61E-05 | 1473 | 1 | 0.61 | 3.23E-02 |
| *EFNB3* | 1.62E-05 | 1028 | 1 | 0.43 | 3.25E-02 |
| *PNLIPRP2* | 1.74E-05 | 1425 | 1 | 0.59 | 3.48E-02 |
| *KRBA2* | 1.79E-05 | 1401 | 1 | 0.58 | 3.59E-02 |
| *SLC26A2* | 2.40E-05 | 2119 | 1 | 0.88 | 4.77E-02 |
| *RNF220* | 2.74E-05 | 1715 | 1 | 0.71 | 5.44E-02 |
| *EEPD1* | 3.02E-05 | 1717 | 1 | 0.71 | 5.98E-02 |
| *TMC2* | 3.52E-05 | 2741 | 1 | 1.14 | 6.92E-02 |
| *HNRNPU* | 4.10E-05 | 2492 | 1 | 1.03 | 8.02E-02 |
| *GRIK3* | 5.26E-05 | 2776 | 1 | 1.15 | 1.02E-01 |
| *ANKRD12* | 6.41E-05 | 5915 | 1 | 2.45 | 1.22E-01 |
| *DNAJC13* | 7.72E-05 | 6787 | 1 | 2.81 | 1.46E-01 |
| *SVEP1* | 1.25E-04 | 10732 | 1 | 4.44 | 2.25E-01 |
